# CT-based peritumoral radiomics nomogram on prediction of response and survival to induction chemotherapy in locoregionally advanced nasopharyngeal carcinoma

**DOI:** 10.1007/s00432-023-05590-5

**Published:** 2024-01-29

**Authors:** Fanyuan Zeng, Zhuomiao Ye, Qin Zhou

**Affiliations:** 1 Department of Oncology, Xiangya Hospital, Central South University, Changsha, 410008 Hunan China; 2 Translational Medicine Research Center (TMRC), School of Medicine, Chongqing University, Shapingba, Chongqing, 400044 China

**Keywords:** Nasopharyngeal carcinoma, Immunotherapy, Chemotherapy, Radiomics, Nomogram

## Abstract

**Purpose:**

The study aims to harness the value of radiomics models combining intratumoral and peritumoral features obtained from pretreatment CT to predict treatment response as well as the survival of LA-NPC(locoregionally advanced nasopharyngeal carcinoma) patients receiving multiple types of induction chemotherapies, including immunotherapy and targeted therapy.

**Methods:**

276 LA-NPC patients (221 in the training and 55 in the testing cohort) were retrospectively enrolled. Various statistical analyses and feature selection techniques were applied to identify the most relevant radiomics features. Multiple machine learning models were trained and compared to build signatures for the intratumoral and each peritumoral region, along with a clinical signature. The performance of each model was evaluated using different metrics. Subsequently, a nomogram model was constructed by combining the best-performing radiomics and clinical models.

**Results:**

In the testing cohort, the nomogram model exhibited an AUC of 0.816, outperforming the other models. The nomogram model’s calibration curve showed good agreement between predicted and observed outcomes in both the training and testing sets. When predicting survival, the model’s concordance index (C-index) was 0.888 in the training cohort and 0.899 in the testing cohort, indicating its robust predictive ability.

**Conclusion:**

In conclusion, the combined nomogram model, incorporating radiomics and clinical features, outperformed other models in predicting treatment response and survival outcomes for LA-NPC patients receiving induction chemotherapies. These findings highlight the potential clinical utility of the model, suggesting its value in individualized treatment planning and decision-making.

**Supplementary Information:**

The online version contains supplementary material available at 10.1007/s00432-023-05590-5.

## Introduction

Nasopharyngeal carcinoma is a relatively rare cancer that affects the upper part of the pharynx located behind the nose. It is more common in Southeast Asia and North Africa(Wei and Sham 2005; Chen et al. 2019). The exact cause is not fully understood, but risk factors such as the Epstein-Barr virus and chemical exposure have been associated with an increased risk(Chua et al. 2016). Symptoms include nosebleeds, difficulty breathing or swallowing, and other related symptoms. LA-NPC is a type of nasopharyngeal carcinoma that has spread to nearby tissues, which poses challenges to current treatment. Current guidelines recommend concurrent chemoradiotherapy (CCRT) based on platinum chemotherapy with or without induction chemotherapy (IC)(Blanchard et al. 2015). Induction chemotherapy, which is administered before the main treatment, has shown promising results in improving treatment success and survival rates in various cancers, including LA-NPC(Zhang et al. 2016; Yang et al. 2019).

The Response Evaluation Criteria in Solid Tumors (RECIST) is a widely adapted tool for assessing the effectiveness of induction chemotherapy in patients with cancer(Eisenhauer et al. 2009) and categorize patients based on tumor response. However, there is a need for accurate measures to predict treatment responses and survival before induction chemotherapy.

In general, Computed Tomography (CT) is widely utilized in clinical settings due to its whole-body scanning capability and high repeatability, as it is the optimal imaging method for many anatomical sites. In addition, CT-guided biopsy can assist in diagnosis, making it an important means of tumor localization, staging, and evaluating treatment efficacy (Patel et al. 2023). It is also a widely used imaging technique for diagnosing and monitoring NPC (Abdel Khalek Abdel Razek and King 2012). Despite MRI enables superior soft tissue characterization and is widely employed for the evaluation of nasopharyngeal carcinoma (King et al. 2019), CT imaging is often the primary imaging technique for radiation therapy planning and dose computation, which mainly offers several advantages in this study. Firstly, CT is more widely available across centers, requires reduced scan time, and entails lower expense than MRI (Junn et al. 2021), thereby facilitating efficient high-volume analysis. In addition, CT provides higher resolution depiction of bone and vascular invasion, (Rumboldt et al. 2006; Nayak et al. 2018) which can be crucial for assessing tumor infiltration and treatment effects. Moreover, CT might be the only option for patients with MRI contraindications (e.g., intracranial metallic foreign bodies, claustrophobia) (Hiyama et al. 2019). On a technical level, CT imaging protocols generally have higher spatial resolution than MRI (Wippold II 2007). Therefore, while MRI excels at soft tissue characterization in many applications, the visualization, availability, and analytical strengths of CT highlighted here demonstrate it can serve as an informative platform for radiomics analysis. Recent advancements in radiomics, which analyze imaging features in a non-invasive and objective manner, have shown promise in predicting outcomes and adverse events in NPC. CT-based radiomics, particularly in the analysis of both intra-tumoral and peritumoral regions, can provide valuable insights into the characteristics of NPC and the tumor microenvironment(Liu et al. 2019; Peng et al. 2019; Daoud et al. 2019; Mayerhoefer et al. 2020).

However, most previous research has focused on MRI-based radiomics, intra-tumoral regions, and a limited number of patients(Zhao et al. 2020; Yongfeng et al. 2021; Cai et al. 2022). There is a lack of studies combining clinical data and imaging features, especially in patients receiving targeted therapy or immunotherapy. Therefore, this study aims to investigate both intra-tumoral and peritumoral regions in CT scans to predict treatment response in LA-NPC patients receiving different classes of therapy. The goal is to develop a user-friendly nomogram that can be applied in clinical settings.

## Methods

### Patients

A total of 276 cases admitted between January 2018 and January 2022 in Xiangya Hospital were enrolled in the study. This study was approved by the Medical Ethics Committee of Xiangya Hospital. Due to its retrospective nature, patient consent was waived. The clinical treatment response after IC was defined as stable disease (SD), progressive disease (PD), partial response (PR), or complete response (CR) based on the Response Evaluation Criteria in Solid Tumors 1.1 (RECIST) criteria. Patients were categorized into responders (CR/PR) and non-responders (SD/PD). The inclusion criteria were (i) nasopharyngeal cancer was confirmed by pathological examination; (ii) complete data of CT recorded less than two weeks before cancer treatment and (iii) classified as III or IVA according to the 8th edition of AJCC. Exclusion criteria were: (i) Received nasopharyngeal carcinoma treatment before CT examination; (ii) accompanied by other tumors; (iii) with incomplete clinical characteristics and (iv) with unqualified data of CT. Randomized sampling was used to divide all cases in the primary cohort into a training and an internal testing group. In the cohort, 221 patients were randomly allocated to the training cohort (142 responders vs 79 non-responders) and 55 patients were allocated to the testing cohort (36 responders vs 19 non-responders), respectively.

### CT scanning parameters

During CT image acquisition, patients were positioned in a supine position, using thermoplastic masks and head-neck-shoulder immobilization devices on a uniform radiotherapy positioning board. The scanning range extended from the top of the head to the tracheal bifurcation. A SIEMENS SOMATOM Definition AS + Spiral CT simulator was used to perform the CT scans for each patient with axial scanning, a tube voltage of 120 kV, a tube current of 200 mA, an exposure value of 250 mAs, an exposure time of 1000 ms. Slice thickness was 3 mm; Slice spacing was 0.98 mm × 0.98 mm × 3 mm, and the dimensions were 512 × 512. CT parameters and body position were consistent for all patients.

### Tumor segmentation and mask dilation

A radiation oncologist with 10 years of work experience contoured the regions of interest (ROIs) slice by slice based on the border of the tumor using Eclipse from Varian Medical Systems (https://www.varian.com). Another senior radiologist with 15 years of experience validated all manual delineations. Registered pretreatment MRI scans were obtained as part of our standard clinical protocol. After initial ROI contouring on CT in ITK-SNAP software, the MRI data was referenced for refinement of ROI determination, leveraging the superior soft tissue resolution. All ROIs were discussed and verified on Varian and ITK-SNAP until a consensus was reached. The collected CT images were stored in the picture archiving and communication system in a DICOM format. Supplementary file 1: Figure S1 shows examples of CT images with delineated ROIs. In addition, the original ROI mask was progressively enlarged with different radial distances outside the tumor at 1 mm intervals (up to a dilation distance of 5 mm) using a “SimpleITK” package in Python version 3.7, to evaluate the prediction performance of peritumoral regions. As a result, 5 new masks representing different peritumoral regions were obtained for each patient. Both the intra-tumoral and peri-tumoral regions were stored in NII(nifti) format and were applied in the analysis.

### Radiomics feature extraction

All the radiomics features are extracted and classified into three categories: (I) geometry, (II) intensity, and (III) texture based on Pyradiomics for each patient. The geometry features describe the tumor’s three-dimensional shape characteristics, while the intensity features characterize the first-order statistical distribution of the tumor’s voxel intensities. Additionally, the texture characteristics describe the patterns or the second and higher-order spatial distributions of intensities. Several methods are used to extract texture features in this case, including the gray-level co-occurrence matrix (GLCM), gray-level run length matrix (GLRLM), gray-level size zone matrix (GLSZM), and neighborhood gray-tone difference matrix (NGTDM). As a result, a total of 3668 features were extracted from the intratumoral and peri-tumoral regions.

### Radiomics feature selection

To reduce irrelevant features, we conducted the Mann–Whitney *U*-test statistical test and feature screening for all radiomics features. Only the *p* value < 0.05 of the radiomics feature was kept. Moreover, for features with high repeatability, Spearman’s rank correlation coefficient was also used to calculate the correlation between features, and one of the features with a correlation coefficient greater than 0.9 between any two features is retained. To retain the ability to depict features to the greatest extent, we used a greedy recursive deletion strategy for feature filtering, in other words, the feature with the greatest redundancy in the current set is deleted each time. The least absolute shrinkage and selection operator (LASSO) regression model was used on the discovery data set for signature construction. Depending on the regulation weight *λ*, LASSO shrinks all regression coefficients towards zero and sets the coefficients of many irrelevant features exactly to zero. To find an optimal *λ*, tenfold cross-validation with minimum criteria was employed, where the final value of *λ* yielded minimum cross-validation error. The retained features with nonzero coefficients were used for regression model fitting and combined into a radiomics signature. Subsequently, we obtained a radiomics score for each patient by a linear combination of retained features weighed by their model coefficients. The Python scikit-learn package was used for LASSO regression modeling.

### Radiomics signature and clinical signature establishment

After Lasso feature screening, we input the final features of intra-tumoral and peri-tumoral regions into the machine learning models including LR(Logistic Regression), SVM(Support Vector Machine), KNN(K-Nearest Neighbors), random forest, extra trees, XGBoost, LightGBM, MLP(Multilayer Perception) for risk model construction. Under this circumstance, we adopt fivefold cross verification to obtain the rad signatures (radiomics signatures) for all tumoral and peri-tumoral regions separately and then compare their prediction performance. After validation of the best-performing dilation distance, we then combined it with an intratumoral region to build an additional radiomics signature.

As for clinical signatures, after inputting numeric clinical data, the feature selection and signature establishment process are similar. To be more specific, the features selected for building clinical signatures were selected by baseline statistics whose *p* value < 0.05. We also used the same machine learning model in radiomics signature-building process. Five-fold cross-validation and test cohort were set to be fixed for a fair comparison. We then compared the prediction performance of all the dilation distances of peri-tumoral regions.

### Radiomics nomogram development

Furthermore, to intuitively and efficiently assess the incremental prognostic value of the radiomics signature and the clinical risk factors, a radiomics nomogram was presented on the testing data set. The nomogram was based on the optimal dilation distance of peri-tumoral regions, together with intra-tumoral regions and clinical signatures. The diagnostic efficacy of radiomics nomogram was tested in the testing cohort, ROC(receiver operating characteristic) curves were drawn to evaluate the diagnostic efficacy of the nomogram. The calibration efficiency of the nomogram was evaluated by drawing calibration curves, and Hosmer–Lemeshow analytical fit was used to evaluate the calibration ability of the nomogram. Furthermore, decision curve analysis (DCA) is used to evaluate the clinical utility of predictive models.

### Model performance in survival analysis

**Fig. 1:**
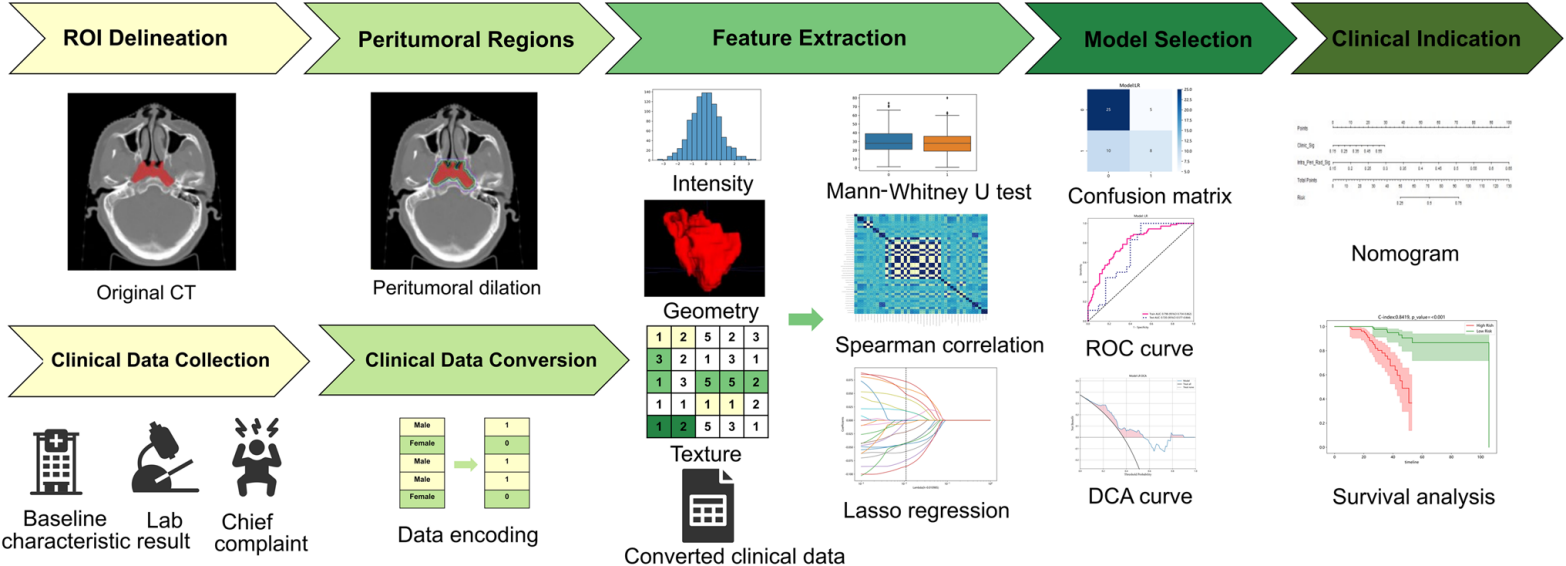
Workflow demonstrating the research design

In the end, Kaplan–Meier curves are employed to assess model performance in survival. Radiomics risk scores and clinical predictors were incorporated as inputs into a Cox proportional hazards model to predict disease-free survival. Patients are separated into high vs low-risk groups based on a threshold of 1.0 on the predicted partial hazards. Concordance index (C-index) was calculated to assess discrimination. The significance of the Kaplan–Meier analysis was determined by using the multivariate log-rank test. To summarize, a flowchart of our research approach is illustrated in Fig. 1. The analysis was performed using various software tools, including Varian Medical Systems, ITK SNAP v.3.8.0, Inkscape v.1.2.1 and custom code written in Python v.3.7.12. Python packages used in the analysis include Pandas v.1.2.4, NumPy v.1.20.2, Seaborn v.0.11.1, Matplotlib v.3.2.2, SciPy v.1.7.3, scikit-learn v.1.0.2, PyRadiomics v.3.0, SimpleITK v.2.1.1, and Lifelines v.0.27.0.

## Results

### Patient characteristics

We compared the clinical characteristics of the patients using an independent sample $t$ test, Mann–Whitney $U$ test, or ${\chi }^{2}$ test, where appropriate. Supplementary file 1: Table S1 showed the baseline clinical characteristics of patients in our cohort respectively. Treatment regimens include induction chemotherapy(e.g., gemcitabine plus cisplatin), targeted therapy(e.g., nimotuzumab), and PD-1/PD-L1 related immunotherapy(e.g., sintilimab). As shown in the table, lymphocyte count, HCG(Human chorionic gonadotropin), transglutaminase, DFS(Disease-free survival) event, DFS time, gender, facial numbness, AJCC(American Joint Committee on Cancer) N staging, histology type, EBV_DNA and treatment category showed a significant difference in the training set, while AJCC N staging and DFS time showed a significant difference in the testing set. There was no significant difference in other characteristics between the testing cohorts.

### Radiomics signature

**Fig. 2:**
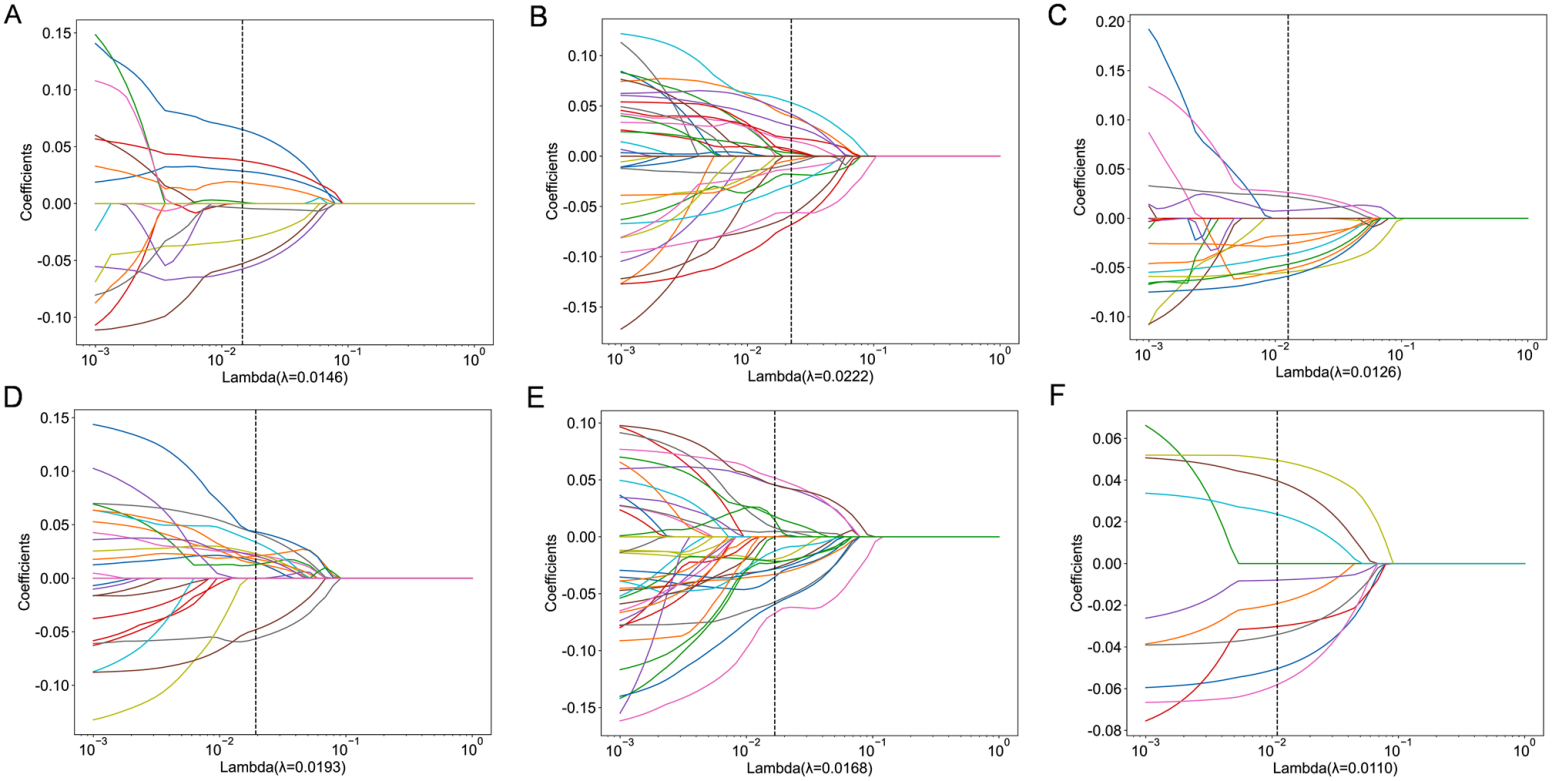
Coefficients of tenfold cross validation for all regions (Subplot A represents intratumoral region, while sublots B-F represent peritumoral dilation region 1–5 mm, respectively)

**Fig. 3:**
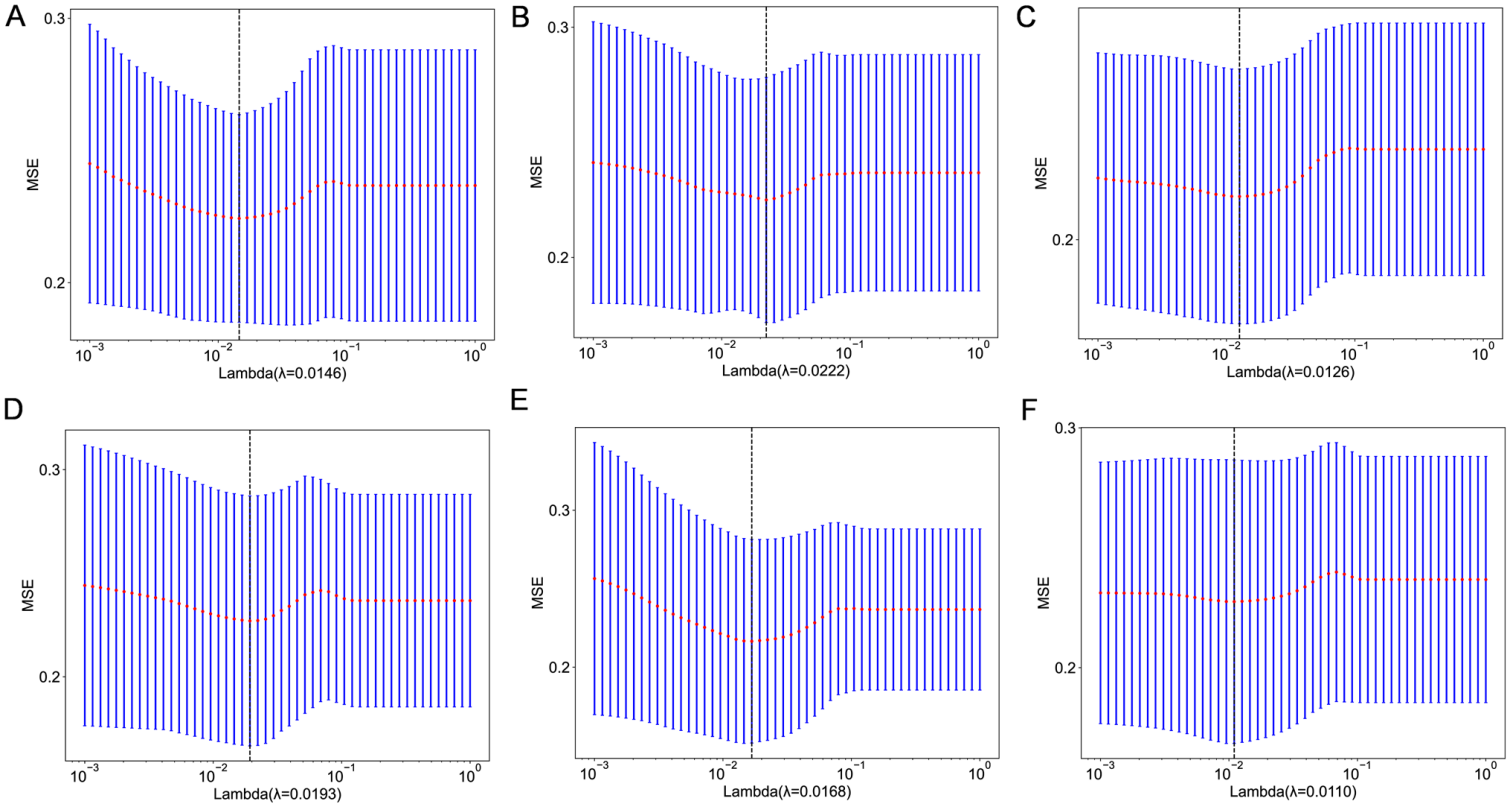
MSE (Mean Standard Error) of tenfold cross validation for all regions(Subplot A represents intratumoral region, while sublots B-F represent peritumoral dilation region 1–5 mm, respectively)

In light of radiomics features, a total of 6 categories, 1834 handcrafted features are extracted, including 360 first-order features, 14 shape features, and 1460 texture features(which includes 440 glcm features, 320 glrlm features, 280 gldm features, 320 glszm features as well as 100 ngtdm features), as shown in Supplementary file 1: Figure S2. All handcrafted features are extracted with an in-house feature analysis program implemented in Pyradiomics(http://pyradiomics.readthedocs.io). Supplementary file 1: Figure S3 shows all features containing all intra-tumoral and peritumoral regions, together with their corresponding p-value results. In addition, nonzero coefficients were selected to establish the Rad score(Radiomics score) with a least absolute shrinkage and selection operator (LASSO) logistic regression model. Coefficients and MSE(mean standard error) of tenfold validation were shown in Figs. 2 and 3.

We applied multiple machine learning models on the features of each intratumoral and peritumoral region, and the performances of all these models from each region are demonstrated in Supplementary file 1: Table S2. We also compared the best prediction performances across all regions, to discover the best dilation distances. As a result, Table 1 exhibited the best prediction performance separately from each region. The results indicated that, in the region of peri-tumor dilation of 2 mm, Extratrees achieved the best value of AUC on the testing cohort, which reached 0.694 for predicting treatment response. Moreover, for intratumoral regions, MLP achieved the best value of AUC on the testing cohort, which reached 0.655 for predicting treatment response.

**Table 1:** Best model performances of each intratumoral and peritumoral region

Dilation	Features	Best_Model	Accuracy	AUC	95%CI	Sensitivity	Specificity	PPV	NPV	Precision	Recall	F1	Threshold	Task
0	9	MLP	0.751	0.772	0.7081–0.8360	0.823	0.711	0.613	0.878	0.613	0.823	0.703	0.355	label-train
0	9	MLP	0.745	0.655	0.4947–0.8153	0.474	0.889	0.692	0.762	0.692	0.474	0.562	0.499	label-test
1	17	XGBoost	1	1	1.0000–1.0000	1	1	1	1	1	1	1	0.487	label-train
1	17	XGBoost	0.618	0.608	0.4532–0.7632	0.632	0.611	0.462	0.759	0.462	0.632	0.533	0.336	label-test
2	10	ExtraTrees	1	1	1.0000–1.0000	1	1	1	1	1	1	1	1	label-train
2	10	ExtraTrees	0.655	0.694	0.5501–0.8373	0.684	0.657	0.5	0.793	0.5	0.684	0.578	0.4	label-test
3	13	RandomForest	0.982	0.998	0.9961–1.0000	0.975	0.986	0.975	0.986	0.975	0.975	0.975	0.5	label-train
3	13	RandomForest	0.636	0.554	0.3920–0.7162	0.368	0.778	0.467	0.7	0.467	0.368	0.412	0.5	label-test
4	18	KNN	0.769	0.824	0.7723–0.8759	0.81	0.746	0.64	0.876	0.64	0.81	0.715	0.4	label-train
4	18	KNN	0.455	0.499	0.3507–0.6463	0.895	0.229	0.378	0.8	0.378	0.895	0.531	0.2	label-test
5	9	LightGBM	0.792	0.878	0.8336–0.9229	0.911	0.725	0.649	0.936	0.649	0.911	0.758	0.34	label-train
5	9	LightGBM	0.473	0.519	0.3637–0.6743	1	0.2	0.396	1	0.396	1	0.567	0.206	label-test

Finally, to fully take advantage of all extracted features to further optimize prediction performance, we combined tumoral regions with the optimal dilation distance(2 mm) to construct the final radiomics signature. Similarly, we also adapted LASSO regression to establish the rad score. Coefficients and MSE of tenfold validation of combined radiomics signature were shown in Supplementary file 1: Figure S4. In the end, a total of 17 features were used, and the final rad score is listed as follows:

rad score = 0.3574660633484163 +  + 0.039752 * intra_exponential_glcm_Imc1 + 0.045907 * intra_exponential_glcm_InverseVariance -0.015887 * intra_exponential_gldm_LargeDependenceHighGrayLevelEmphasis + 0.030417 * intra_exponential_ngtdm_Busyness + 0.019104 * intra_lbp-3D-k_glcm_Imc2 + 0.039637 * intra_lbp-3D-k_glrlm_LongRunHighGrayLevelEmphasis + 0.020697 * intra_original_shape_Flatness -0.046173 * intra_wavelet-LLH_firstorder_Range + 0.014154 * peri_lbp-3D-k_gldm_SmallDependenceLowGrayLevelEmphasis -0.047785 * peri_lbp-3D-k_glszm_ZoneVariance + 0.044680 * peri_lbp-3D-m2_glcm_Idn + 0.033087 * peri_original_glrlm_RunLengthNonUniformityNormalized -0.038392 * peri_wavelet-HHL_firstorder_Mean -0.054844 * peri_wavelet-HHL_glcm_ClusterShade -0.066851 * peri_wavelet-LLH_firstorder_Skewness -0.021093 * peri_wavelet-LLH_glcm_Idmn -0.027197 * peri_wavelet-LLL_ngtdm_Coarseness.

**Fig. 4:**
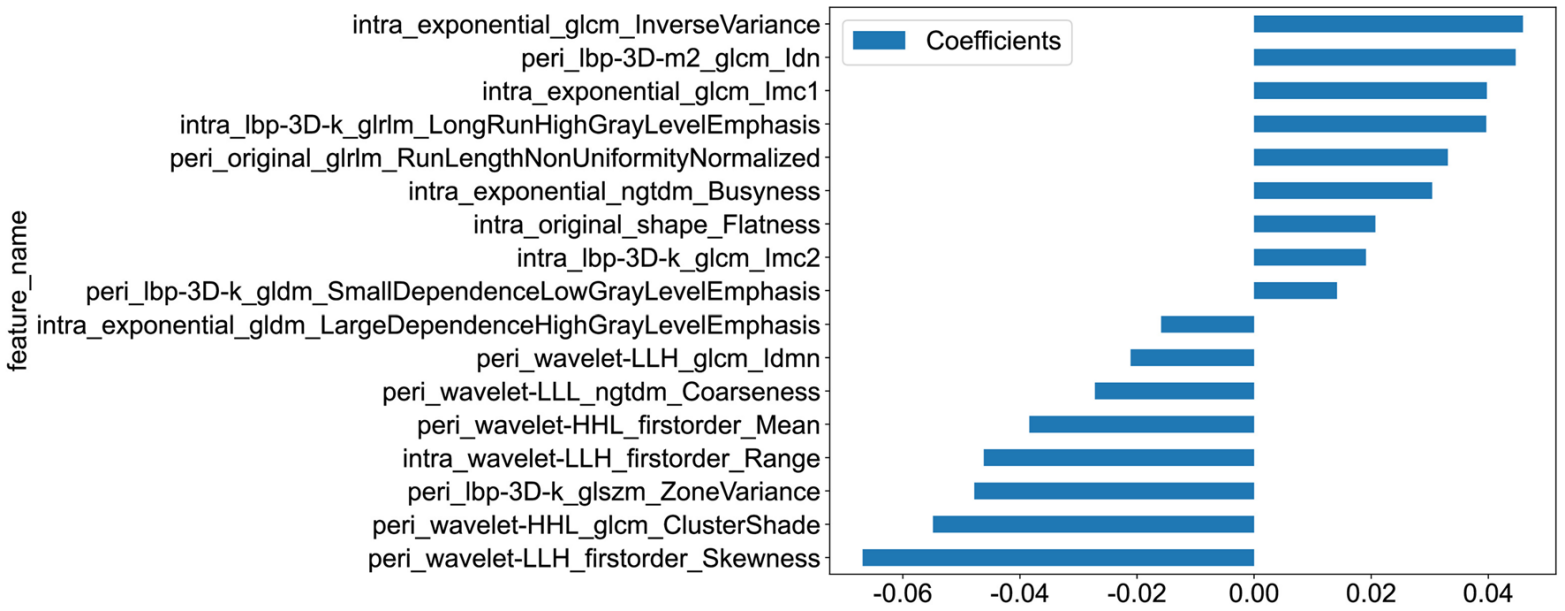
The histogram of the Rad-score based on the selected features in combined radiomics model

**Fig. 5:**
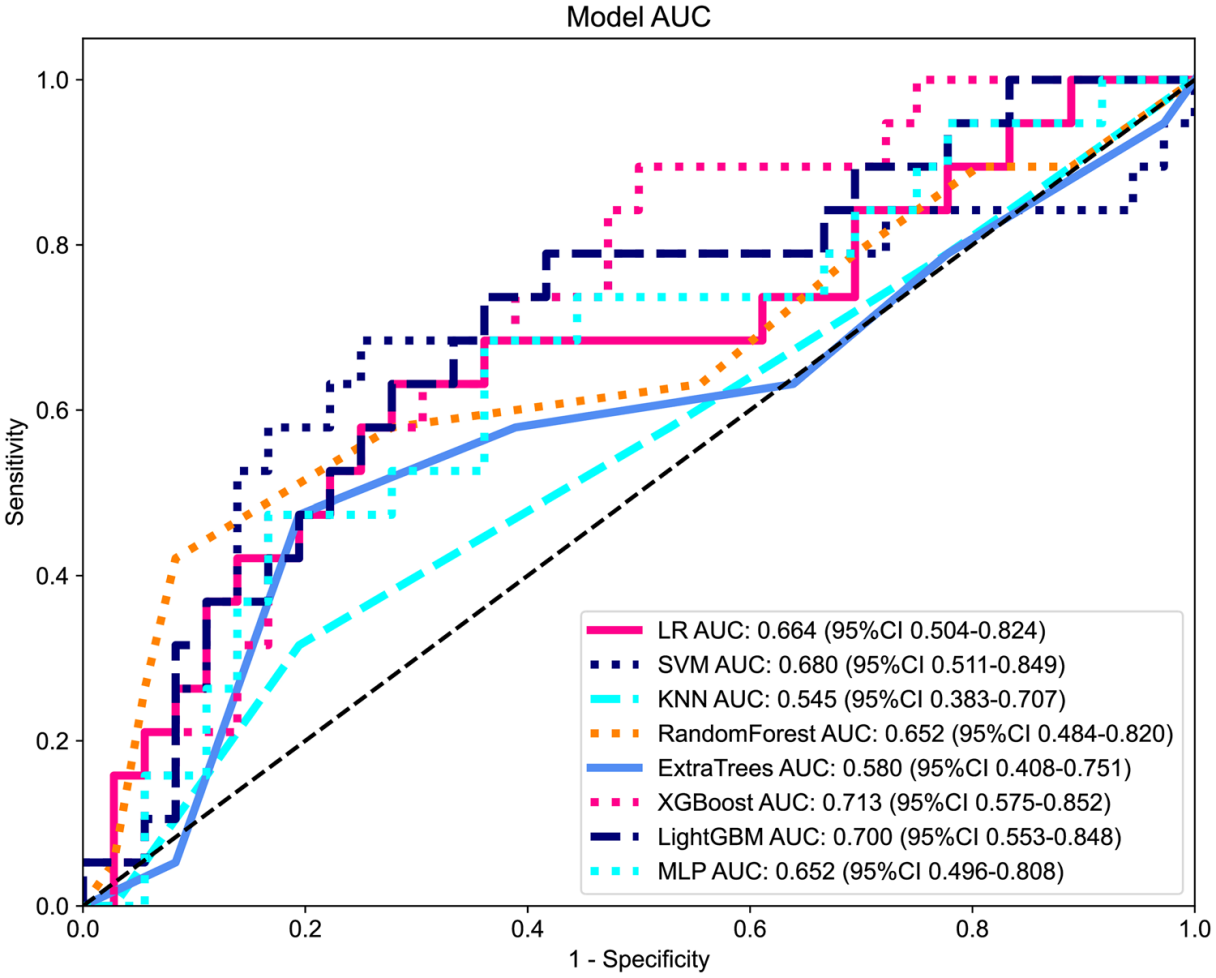
Model performances of combined radiomics signature

The coefficients value in the final selected non-zero features in the radiomics model are shown in Fig. 4. Like previous procedures, multiple machine learning models were utilized, and the ROC curves of these models in the testing cohort are demonstrated in Fig. 5. As shown in the figure, XGBoost achieved the best result, with an AUC of 0.713(95%CI 0.575–0.852), with LightGBM coming second, with an AUC of 0.700(95%CI 0.553–0.848). To better understand the prediction performance in the testing cohort, the prediction score histogram, as well as the confusion matrix of the two models from combined radiomics signature is shown in Supplementary file 1: Figure S5.

#### Clinical signature

**Fig. 6:**
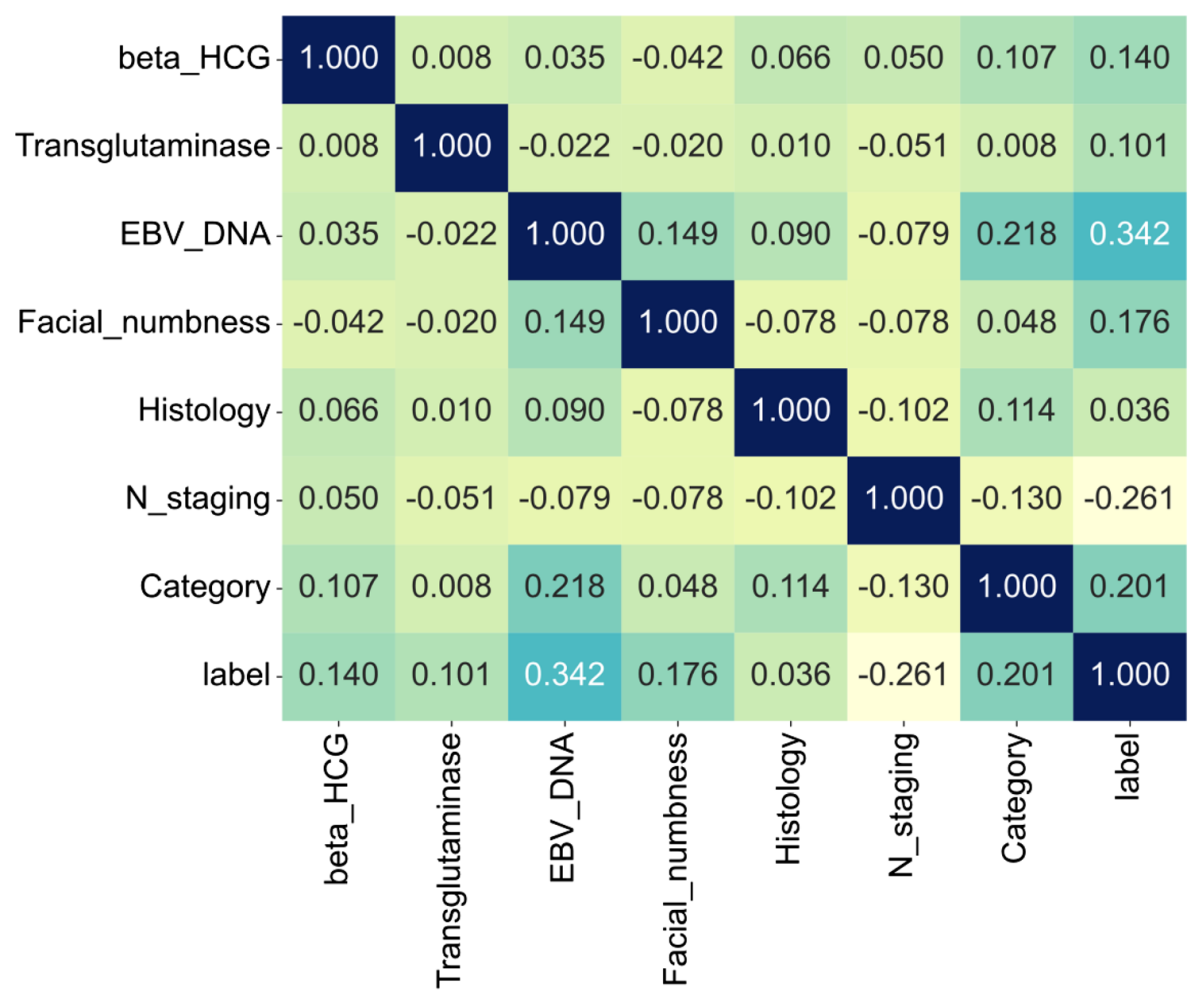
Spearman correlation coefficients of each clinical feature

As for the clinical signature, Fig. 6 shows the correlations between each clinical feature, which indicates that EBV-DNA has a maximum correlation coefficient. Clinical signatures of patients were calculated in a similar way to radiomics signature. In the end, clinical features of *β*-HCG, transglutaminase, EBV-DNA, facial numbness of patient complaint, histology, N staging, and treatment category are included. After the features were selected, multiple machine learning models were also adapted as well as their prediction performances were evaluated. The details of all models’ performances are shown in Supplementary file 1: Table S4. Moreover, the ROC curves of models in the testing cohort were displayed in Supplementary file 1: Figure S6.

#### Radiomics nomogram

**Fig. 7:**
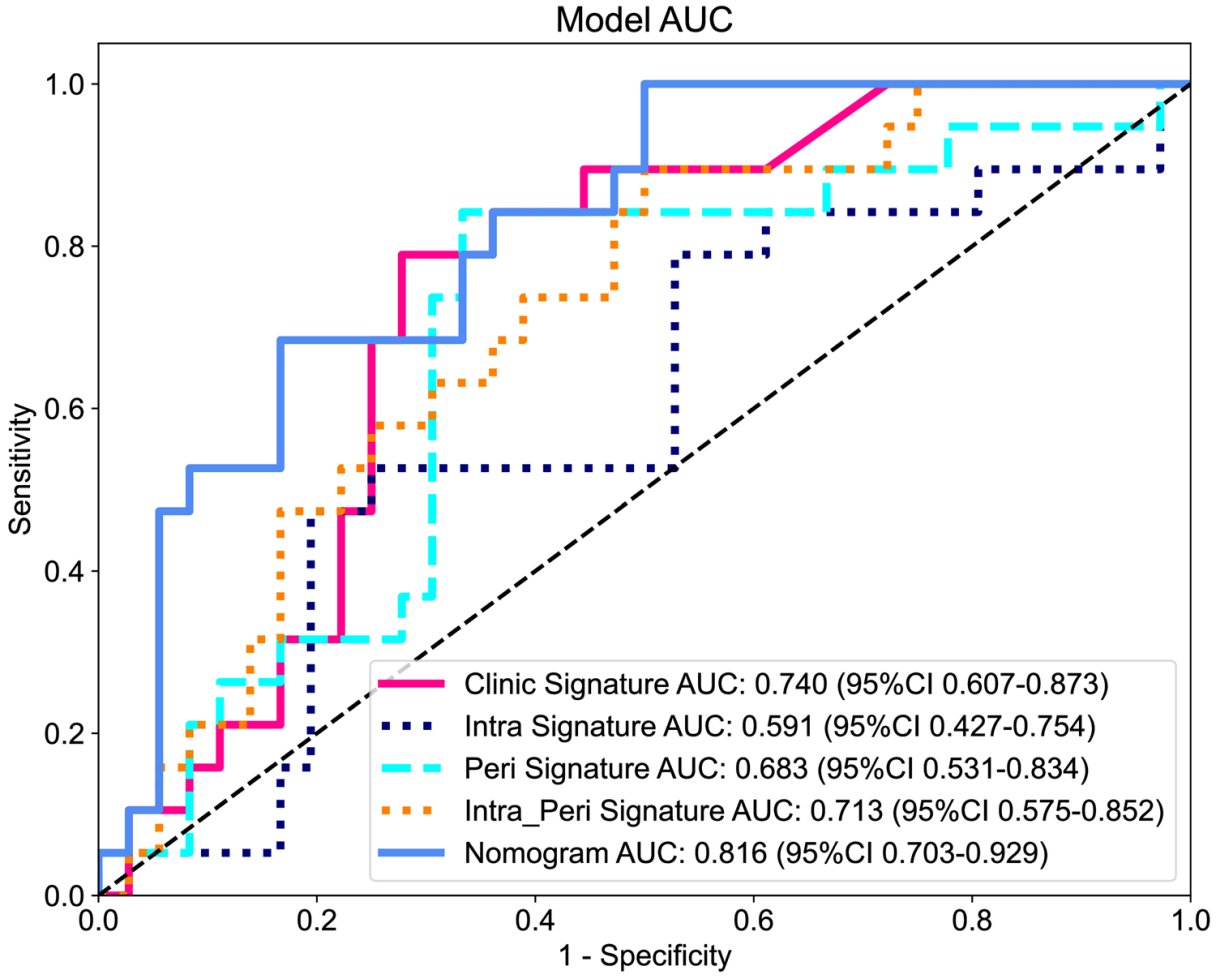
ROC curves of clinical as well as radiomics signatures

According to performances in the combined radiomics signature and clinical signatures, XGBoost algorithm was performed to combine the clinical signature and rad signature to establish the nomogram. To further compare the clinical signature and rad signature and nomogram, the Delong test was used. The performance metrics as well as comparison results were shown in Table 2, Supplementary file 1: Table S4 and Fig. 7. As were shown in the results, in training cohort, both clinical signature and peri-tumoral signature get a great fitting. In the testing cohort, clinical signature and nomogram showed great performance. Overall, the nomogram showed the best performances, and it is statistically better than the intratumoral radiomics model according to delong’s test.

**Table 2:** Indicators of different models

Signature	Accuracy	AUC	95% CI	Sensitivity	Specificity	PPV	NPV	Precision	Recall	F1	Threshold	Cohort
Clinic Signature	0.968326	0.988857	0.9768–1.0000	0.936709	0.985915	0.973684	0.965517	0.973684	0.936709	0.954839	0.525636	Train
Intra Signature	0.9819	0.995275	0.9890–1.0000	0.962025	0.992958	0.987013	0.979167	0.987013	0.962025	0.974359	0.397832	Train
Peri Signature	0.995475	0.998752	0.9962–1.0000	1	0.992958	0.9875	1	0.9875	1	0.993711	0.520984	Train
Intra_Peri Signature	1	1	1.0000–1.0000	1	1	1	1	1	1	1	0.486744	Train
Nomogram	1	1	1.0000–1.0000	1	1	1	1	1	1	1	0.640543	Train
Clinic Signature	0.745455	0.739766	0.6070–0.8726	0.789474	0.742857	0.6	0.866667	0.6	0.789474	0.681818	0.391791	Test
Intra Signature	0.690909	0.590643	0.4274–0.7539	0.473684	0.828571	0.5625	0.74359	0.5625	0.473684	0.514286	0.480967	Test
Peri Signature	0.727273	0.682749	0.5313–0.8342	0.842105	0.666667	0.571429	0.888889	0.571429	0.842105	0.680851	0.332525	Test
Intra_Peri Signature	0.636364	0.71345	0.5749–0.8520	0.894737	0.514286	0.485714	0.9	0.485714	0.894737	0.62963	0.197014	Test
Nomogram	0.781818	0.815789	0.7029–0.9287	0.684211	0.833333	0.68421	0.833333	0.68421	0.684211	0.684211	0.387917	Test

We also constructed calibration curves to evaluate model performances. Supplementary file 1: Figure S7 depicts the calibration curves in the training and testing cohort. Nomogram calibration curves show good agreement between predicted and observed prediction of treatment response in training and test cohort. The *P* values of the Hosmer–Lemeshow test of clinical signature, radiomics signature and nomogram are shown in Supplementary file 1: Table S5. The *p*-value of the nomogram of the Hosmer–Lemeshow test is larger than 0.05, which generally shows that the nomogram fits great in both the training and test cohort.

**Fig. 8:**
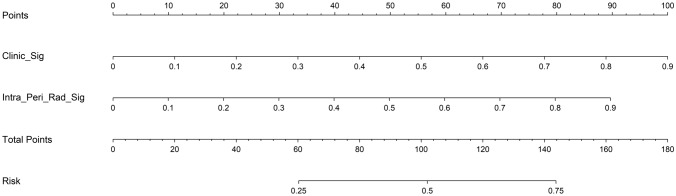
A nomogram based on the combined radiomcis signature as well as a clinical signature to predict treatment response

In this study, we also evaluated each model through decision curve analysis(DCA). The decision curves for the clinical signature, rad signature and radiomics nomogram are presented in Supplementary file 1: Figure S8. Compared with scenarios in which no prediction model would be used (ie, treat-all or treat-none scheme), radiomics nomogram showed benefit for intervention in patients with a prediction probability compared to the clinical signature, intratumoral signature, peritumoral signature, as well as combined signature when threshold probability is at 0.2–0.25, 0.4–0.8. Treatment response prediction using radiomics nomogram has been shown to have better clinical benefits. Finally, Fig. 8 shows the nomogram for clinical use in predicting treatment response.

#### Survival analysis of the model

**Fig. 9:**
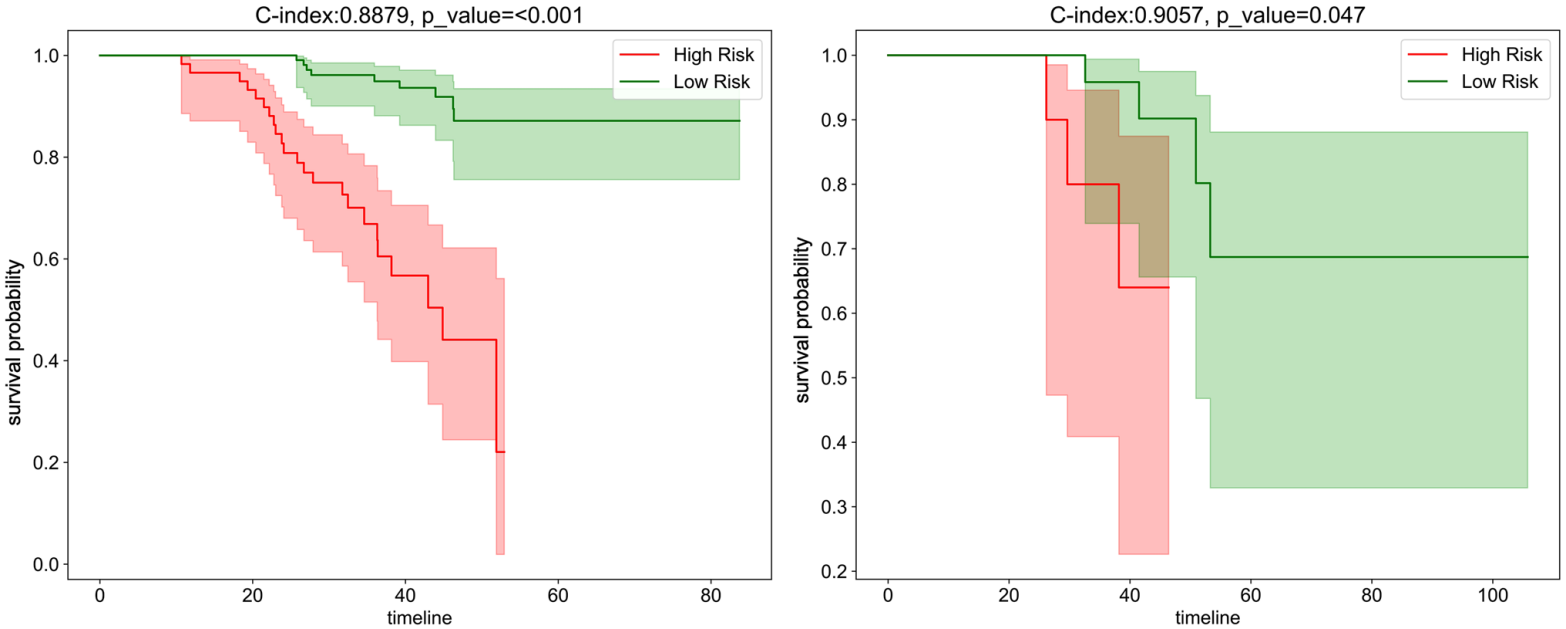
Kaplan–Meier plots (Left: Training cohort. Right: Testing cohort, timeline units: months)

**Fig. 10:**
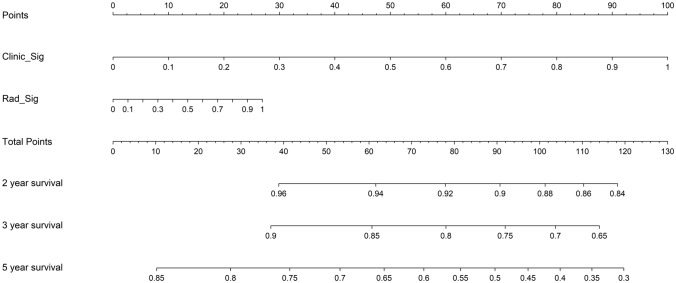
Nomogram for evaluating patient survival

After incorporating radiomics risk scores and clinical predictors into a Cox regression model, we employed it to stratify patients by predicted disease-free survival outcomes. The nomogram-cox model achieves strong discrimination in both training and testing cohorts, indicated by concordance index values of 0.89 and 0.90 respectively (Supplementary file 1: Table S6). in both cohorts. We then tested the models and performed Kaplan–Meier (KM) analysis to validate clinical utility. It revealed significantly differentiated survival curves, with log-rank *p*-values < 0.05 in both cohorts, as shown in Fig. 9. This indicates the variables driving risk predictions are associated with actual underlying disease aggressiveness. To facilitate clinical applications, we also constructed a nomogram for the models to predict patient survival outcomes, as seen in Fig. 10. The constructed nomogram provides an interactive interface to parse how radiomic and clinical information in concert inform survival projections for a given patient.

## Discussion

Despite the fatality of nasopharyngeal carcinoma, the implementation of immunotherapy and targeted therapy has dramatically changed the anti-tumor strategies. A recent report demonstrated that PD-1/PD-L1 blockers or EGFR-targeted drugs combined with induction chemotherapy can substantially improve patient outcomes(Zhang et al. 2018). However, only a portion of the population responds to immunotherapy or targeted therapy(Chan et al. 2005; Hsu et al. 2017), which calls for effective measures that predict responses for these treatments. Despite theoretically PD-1 expression on tumor cells can predict treatment response of immunotherapy, but there are inaccordances of previous research(Wu et al. 2019; Litchfield et al. 2021). Recently, a study revealed the heterogeneity of tumor microenvironment (TME) via virtual microdissection of gene expression profiles, which classified patients into 3 subgroups(Chen et al. 2021), though it did not utilize the medical imaging generated from each patient. Another study demonstrated the potential significance of peritumoral regions of MRI in the treatment response of lymphatic node metastasis(Xu et al. 2023). Despite encouraging results, the study cohort only recruited 145 patients and only explored 2 mm dilation, reaching an AUC of 0.78 in the clinic combined with radiomics model in the testing set.

Both CT and MRI are crucial tools in diagnosing and staging NPC. While MRI excels at soft tissue characterization, the strengths of CT in bone visualization and pragmatic factors including wider accessibility, rapid scan time demonstrate CT can also yield informative imaging phenotypes through a radiomics approach. In this study, we have collected CT images and evaluated intratumoral and peritumoral regions from patients receiving multiple kinds of treatment for the prediction of induction chemotherapy response in nasopharyngeal carcinoma. The radiomics signature that is derived from a 2 mm dilation distance achieves the best prediction performance, compared across all tumoral and peri-tumoral regions. The results indicate that cancer cells tend to migrate to adjacent regions, which changes the textural, intensity or shape features of medical imaging, as validated by previous analyses(Shi et al. 2022). Such phenomenon has been observed in other tumor/disease types, such as cervical cancer and lung cancer (Zhuo et al. 2020). Our study, however, demonstrates that the peritumoral region of nasopharyngeal carcinoma can provide valuable insight into predicting treatment response, including patients receiving immunotherapy or targeted therapy, which previous research has merely addressed.

First, we extracted a total of 1834 features from CT image from each patient. After selecting the optimal dilation distance and combining it with intratumoral regions, 17 features were kept in building the final combined radiomics signature. In these features, 8 are intra-tumoral and 9 are peri-tumoral, which may indicate that since peri-tumoral regions contain the micro-environment of the tumor, they can be valuable in determining the outcome of treatment. Considering feature categories, most of the features in the radiomics signature are GLCM(gray-level co-occurrence matrix) features. The GLCM is calculated by counting the number of times different gray-level values occur at specific spatial relationships within an image which describes how image intensities are spatially correlated(Parekh and Jacobs 2016). GLCM-based features are widely employed textural features in various medical imaging modalities to analyze and describe biological tissue of interest. Moreover, there are 3 first-order, 2 gldm, 2 glrlm, 2 ngtdm, 1 glszm and 1 shape features utilized in the radiomics signature, ordered by numerical counts. In summary, most features in the combined radiomics score are combined texture features, which are higher-dimensional features that cannot be calculated from direct inspection of the image, which neccessitates the establishment of radiomics signature.

Our generated combined radiomics signature from intratumoral and peritumoral regions were cross-validated by several common machine learning algorithms, and XGBoost achieved the highest score in the testing cohort, with an AUC of 0.713(0.575–0.852, 95%CI). XGBoost leverages gradient boosting, an ensemble learning technique, to iteratively build a series of weak prediction models and effectively combine them to create a strong predictive model. This iterative process allows XGBoost to continuously improve predictions and achieve higher accuracy(Chen and Guestrin 2016). For clinical features, some clinical signatures have been related to the response to induction therapy. According to previous research, plasma EBV-DNA levels, age, sex, T stage as well as the overall clinical stage can be used to predict the outcome of patients receiving induction chemotherapy(Liao et al. 2022). Histology of NPC is also a well-known factor contributing to patient response(Colaco et al. 2013). However, in our study, we discovered that more clinical features such as HCG(human chorionic gonadotropin), transglutaminase as well as facial numbness of patient symptoms could be connected to the outcome of induction chemotherapy. According to previous research, HCG has been reported to exhibit a transient rise in cancer patients undergoing induction chemotherapy(Lo et al. 2000). It was previously reported that transglutaminase-2 mediated chemotherapy resistance in nasopharyngeal squamous cell carcinoma cells, thus promoting cancer progression(Kim et al. 2011). Additionally, symptoms include facial numbness could be caused by infiltration of enlarging tumor towards peripheral structures, such as cranial nerves and bony structures, which indicates a poorer prognosis. Overall, the clinical signature we adapted in this study reached an AUC of 0.740(0.607–0.873,95%CI) of a testing cohort in our study. While β-HCG and transglutaminase have not been adequately standardized for clinical NPC management in contrast to widely used indicators like EBV-DNA, our intention was discovery-oriented assessment of their potential utility for incorporation into ongoing validation trials and eventual translation. Despite further research needed to determine the significance of these clinical features, they serve as potential candidates for future therapy targets.

Combining radiomics signature and clinical signature, we develop a handy nomogram that can be used in clinical scenarios. The nomogram has achieved prediction performance with an AUC of 1.00(1.00–1.00, 95%CI) in the training cohort and 0.816(0.702–0.928, 95%CI) in the testing cohort, which is the highest performing model in each cohort. The result is comparable to another recent study that predicted induction chemotherapy response using transfer learning, which achieved an AUC of 0.811 in the testing cohort(Yang et al. 2022). We also evaluated the performance of nomogram with other models using ROC curve, calibration curves and decision curve analyses. The potential clinical value of our nomogram was validated by the decision curve analysis, which displayed that using our nomogram can obtain more net benefit for patients. Moreover, in our study, the target variable is a combined label: responder(CR/PR) vs non-responder(SD/PD), which is a grouping strategy adapted by multiple studies(Wang et al. 2023, 2021). This approach streamlines model development and evaluation by enabling a binary classification approach, which is a simpler model than multi-class classification and could lead to better model performances.

In addition, we also tested the nomogram model’s performance with the survival data from our cohort. We use DFS (Disease-free survival) as an endpoint in the survival analysis. Disease-free interval is defined from the start of the study (often the start of treatment) to the recurrence of the disease or death from any cause in retrospective studies(Robinson et al. 2014) which provides valuable insights into the effectiveness of the treatment in preventing disease recurrence. Recent studies reported that DFS could serve as a surrogate endpoint for OS (overall survival) in several cancers, including head and neck cancers (George et al. 2018; Savina et al. 2018). In terms of survival prediction, the model reaches a C-index of 0.9057(*p* < 0.05) in the testing cohort. We also construct a nomogram for evaluating patient survival in clinical use. The nomogram displays great utility in predicting patient survival apart from treatment response, which facilitates individualized treatment of LA-NPC patients.

Our study also has some limitations. First, the lack of overall survival analysis, the gold standard for evaluating treatment efficacy. Primarily, the short follow-up period provided inadequate statistical power to establish and validate an overall survival model. While disease-free survival provides an interim measure of outcome, claims of clinical utility require confirming an ability to predict overall survival advantage. Second, all ROI were manually contoured, which could be prone to subjective errors. Automatic segmenting techniques should be applied to reduce biases. In addition, this investigation only included CT images. Since a recent report has investigated that molecular landscaping was able to stratify patient subtypes(Ding et al. 2021), integrating more types of patient data (such as MRI, genomic data, etc.) could be more comprehensive and lead to better-predicting performances. Finally, before the clinical deployment of our model, further prospective testing in new patient cohorts is necessary to evaluate model performance comparing predictions to eventual real-world patient outcomes across centers. Assessment of general clinical utility was not feasible within our single center, retrospective design, which has inherent limitations related to potential selection biases and restricted generalizability.

## Conclusions

We have made significant progress in developing radiomics models using intratumoral and peritumoral regions of CT images in LA-NPC patients. Furthermore, by integrating a clinical model that incorporates relevant clinical features, we have successfully created a comprehensive nomogram model. This nomogram model has demonstrated remarkable performance in predicting treatment response and survival outcomes for patients undergoing various types of induction chemotherapies. The model holds great promise for advancing individualized treatment approaches for patients with LA-NPC. By leveraging the power of radiomics and clinical data, our model offers valuable insights into treatment planning and decision-making. This enables healthcare professionals to tailor treatments to the specific needs of each patient, ultimately leading to improved outcomes and enhanced patient care.

### Supplementary Information

Below is the link to the electronic supplementary material.

Supplementary file1 (DOCX 7723 KB)

## Data Availability

The datasets generated during and/or analyzed during the current study are available from the corresponding author upon reasonable request.
