# Supplementary material for: CT-based peritumoral radiomics nomogram on prediction of response and survival to induction chemotherapy in locoregionally advanced nasopharyngeal carcinoma
Source: J Cancer Res Clin Oncol. 2024 Jan 29;150(2):50. doi: 10.1007/s00432-023-05590-5 (PMC10824876; doi:10.1007/s00432-023-05590-5)
Supplement: Supplementary file 1 — Supplementary file1 (DOCX 7723 KB) [file 432_2023_5590_MOESM1_ESM.docx]

CT-based peritumoral radiomics nomogram on prediction of response and survival to induction chemotherapy in nasopharyngeal carcinoma (Supplementary)

Fanyuan Zeng^*,1^, Zhuomiao Ye^*,1,2^, and Qin Zhou^1,✉^

^*^ These authors contributed equally to this work.

^1^ Department of Oncology, Xiangya Hospital, Central South University, Changsha, Hunan, 410008, China
^2^ Translational Medicine Research Center (TMRC), School of Medicine, Chongqing University, Shapingba, Chongqing, 400044, China.

^✉^ Correspondence: [Qin Zhou <zhouqin5796@csu.edu.cn>](mailto:zhouqin5796@csu.edu.cn)

| 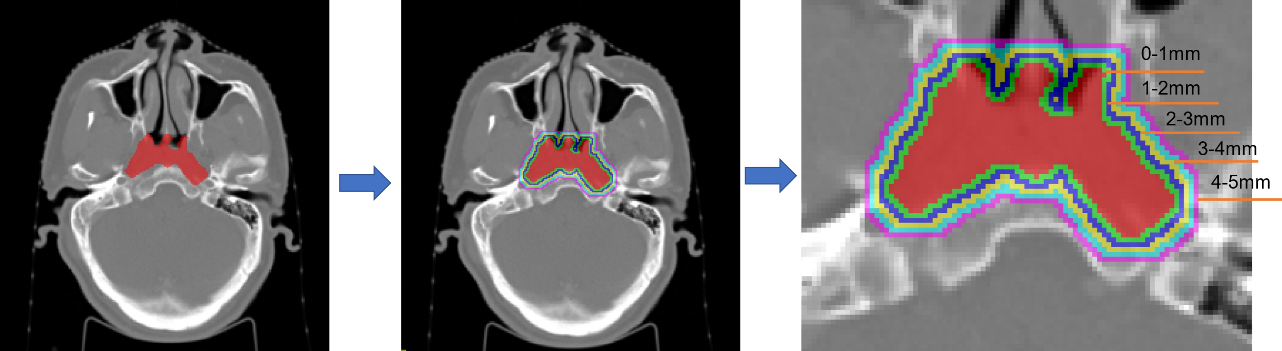  Figure S1: **Original intratumoral region and peritumoral dilations** |
| --- |

Table S1: **Full baseline characteristics of patients in cohorts**

| feature_name | train-label=ALL | train-label=0 | train-label=1 | pvalue | test-label=ALL | test-label=0 | test-label=1 | pvalue |
| --- | --- | --- | --- | --- | --- | --- | --- | --- |
| DFS(disease free survival) time(months) | 39.53±13.84 | 42.11±14.63 | 34.90±10.92 | <0.001 | 40.12±13.74 | 44.51±13.90 | 31.79±8.90 | <0.001 |
| Age(years) | 48.64±10.79 | 48.57±10.60 | 48.77±11.18 | 0.8943657621162255 | 47.07±10.50 | 45.61±10.77 | 49.84±9.66 | 0.15734476316722404 |
| White blood cell count(10^9^/L) | 6.35±2.16 | 6.20±1.83 | 6.63±2.65 | 0.14955694177940757 | 6.46±1.84 | 6.15±1.57 | 7.06±2.20 | 0.08018882094920646 |
| Hemoglobin(g/L) | 138.42±19.28 | 136.63±20.85 | 141.65±15.67 | 0.0637820154619294 | 140.55±11.63 | 141.03±12.88 | 139.63±9.03 | 0.676004648020123 |
| Height(cm) | 164.27±7.48 | 163.55±7.81 | 165.57±6.70 | 0.05408164265144836 | 164.36±8.71 | 164.22±8.34 | 164.63±9.59 | 0.8701042142679182 |
| Weight(kg) | 64.06±10.92 | 63.84±11.22 | 64.47±10.40 | 0.6818271139506046 | 64.54±12.52 | 65.71±12.94 | 62.32±11.68 | 0.3438816954534345 |
| Surface area(m^2^) | 1.79±0.17 | 1.78±0.17 | 1.80±0.16 | 0.39411821553828885 | 1.79±0.20 | 1.81±0.20 | 1.77±0.19 | 0.4837955159291891 |
| Smoking(years) | 9.29±12.56 | 8.39±12.04 | 10.92±13.37 | 0.15056079071379017 | 10.69±14.61 | 8.69±12.26 | 14.47±18.02 | 0.16496451245616262 |
| Drinking(years) | 4.97±10.93 | 5.54±11.69 | 3.96±9.40 | 0.30623201292636226 | 4.67±11.45 | 3.81±9.50 | 6.32±14.61 | 0.4445443273137233 |
| Lymphocyte count(10^9^/L) | 1.52±0.50 | 1.47±0.46 | 1.61±0.56 | 0.04128289851946125 | 1.99±2.82 | 1.52±0.47 | 2.89±4.70 | 0.08617051567111583 |
| Eosinophil count(10^9^/L) | 0.15±0.46 | 0.16±0.56 | 0.14±0.13 | 0.7766896219051038 | 0.16±0.12 | 0.14±0.08 | 0.19±0.16 | 0.12796311234051852 |
| Basophil count(10^9^/L) | 0.03±0.12 | 0.04±0.15 | 0.02±0.03 | 0.19453351726629944 | 0.02±0.04 | 0.02±0.03 | 0.03±0.04 | 0.3575766977233872 |
| Eosinophil ratio(%) | 1.93±1.64 | 1.79±1.32 | 2.18±2.09 | 0.08995859278752455 | 2.52±1.68 | 2.36±1.43 | 2.83±2.08 | 0.32254806132612335 |
| Monocyte count(10^9^/L) | 1.23±2.23 | 1.42±2.50 | 0.88±1.61 | 0.08669475180127129 | 1.07±2.07 | 1.14±2.21 | 0.95±1.82 | 0.751971318804305 |
| Procalcitonin(ng/ml) | 0.20±0.06 | 0.20±0.05 | 0.20±0.07 | 0.6603899778102115 | 0.21±0.05 | 0.20±0.05 | 0.21±0.05 | 0.7278902772568343 |
| CEA(carcinoembryonic antigen)(ng/ml) | 1.92±1.36 | 1.91±1.55 | 1.93±0.91 | 0.9163160941330666 | 5.62±26.96 | 7.62±33.30 | 1.83±1.07 | 0.4533678335508229 |
| AFP(alpha fetoprotein)(ng/ml) | 2.69±1.41 | 2.65±1.28 | 2.76±1.63 | 0.6002408763422105 | 2.86±1.61 | 2.90±1.75 | 2.80±1.36 | 0.8401581201386192 |
| NSE(neuron-specific enolase) (ng/ml) | 11.78±5.13 | 11.40±5.42 | 12.47±4.52 | 0.1387032766918588 | 11.51±4.73 | 11.76±4.24 | 11.03±5.64 | 0.5907626155452348 |
| CYFRA21-1(cytokeratin 19 fragment)(ng/ml) | 7.70±8.31 | 6.97±5.67 | 9.02±11.57 | 0.0775680950248378 | 8.50±7.47 | 7.75±7.45 | 9.93±7.50 | 0.30692565035793135 |
| CA125(cancer antigen 125)(U/ml) | 12.12±5.34 | 12.31±4.81 | 11.78±6.20 | 0.48536861704096224 | 15.63±19.86 | 14.40±15.14 | 17.97±27.01 | 0.531251640822756 |
| CA242(cancer antigen 242)(U/ml) | 6.12±3.45 | 6.24±3.72 | 5.90±2.93 | 0.4829637979020446 | 5.30±2.00 | 5.50±1.97 | 4.91±2.06 | 0.3044108744393213 |
| β-HCG(beta-human chorionic gonadotropin)(U/ml) | 0.31±0.15 | 0.29±0.14 | 0.33±0.17 | 0.04210130871037314 | 0.38±0.75 | 0.42±0.92 | 0.30±0.13 | 0.5930176674324976 |
| Albumin(g/L) | 42.12±2.01 | 42.08±1.86 | 42.18±2.27 | 0.7368942668677617 | 42.37±1.96 | 42.30±1.63 | 42.49±2.51 | 0.7411229172684428 |
| Albumin/Globulin ratio(%) | 1.77±3.98 | 1.67±3.58 | 1.95±4.63 | 0.6210956279491913 | 1.39±0.26 | 1.37±0.29 | 1.42±0.22 | 0.5670327031791875 |
| Total bilirubin(g/L) | 10.00±4.35 | 9.81±4.42 | 10.36±4.24 | 0.36508594958614393 | 9.69±3.93 | 10.08±4.05 | 8.96±3.68 | 0.3212822169561748 |
| Direct bilirubin(g/L) | 4.76±2.01 | 4.59±1.95 | 5.06±2.10 | 0.09086970496942537 | 4.44±1.66 | 4.43±1.58 | 4.47±1.84 | 0.9237725214901413 |
| Uric acid(μmol/L) | 335.89±91.89 | 343.97±93.65 | 321.38±87.32 | 0.07983930072805429 | 349.40±100.49 | 361.03±111.35 | 327.38±73.52 | 0.2412082641255542 |
| Triglycerides(mmol/L) | 1.84±1.07 | 1.82±0.81 | 1.88±1.42 | 0.7009754931614072 | 1.93±1.20 | 2.03±1.30 | 1.73±0.96 | 0.37368463072272184 |
| Cholesterol(mmol/L) | 5.07±1.02 | 5.06±0.94 | 5.11±1.16 | 0.7315729176550554 | 4.80±0.62 | 4.78±0.58 | 4.84±0.71 | 0.7389382223635943 |
| LDH(lactate dehydrogenase)(U/L) | 134.65±154.71 | 144.77±179.38 | 116.46±93.93 | 0.19294665536621447 | 128.87±99.11 | 127.25±96.30 | 131.94±106.88 | 0.8691023460518525 |
| Creatine kinase(U/L) | 87.90±51.83 | 87.10±49.09 | 89.32±56.72 | 0.7606484718907507 | 91.86±36.58 | 96.47±37.12 | 83.12±34.79 | 0.2011892545923806 |
| Chlorium(mmol/L) | 102.05±6.75 | 102.50±2.30 | 101.24±10.87 | 0.18435101002849905 | 102.62±2.32 | 102.76±2.59 | 102.36±1.73 | 0.5473141702242117 |
| ALP(Alkaline phosphatase)(U/L) | 83.16±20.83 | 81.43±21.03 | 86.28±20.23 | 0.09747702893691423 | 84.99±23.85 | 85.54±23.17 | 83.95±25.71 | 0.8169011173126732 |
| Transglutaminase(U/L) | 31.87±26.64 | 28.91±14.87 | 37.20±39.46 | 0.026419673888692874 | 34.14±22.25 | 35.94±26.04 | 30.71±12.13 | 0.4120679148487607 |
| ASTm(mitochondrial aspartate aminotransferase)(U/L) | 7.62±6.68 | 7.15±2.70 | 8.47±10.56 | 0.1623169049076153 | 8.09±4.79 | 8.54±5.77 | 7.22±1.69 | 0.3352504420599687 |
| AFU(alpha-l-fucosidase)(U/L) | 25.68±6.95 | 25.12±6.85 | 26.70±7.04 | 0.10446997899467018 | 25.51±6.45 | 25.88±6.50 | 24.80±6.47 | 0.562712543155077 |
| EBV-DNA(copy) | 2265.72±6286.16 | 1176.11±2905.62 | 4224.25±9494.57 | <0.001 | 1519.96±2834.08 | 1076.08±1512.51 | 2360.99±4301.99 | 0.11055506575252186 |
| Fibrinogen(g/L) | 4.72±15.81 | 5.19±19.72 | 3.86±0.94 | 0.5512464444333621 | 3.65±1.04 | 3.47±1.14 | 4.00±0.74 | 0.07033147302150534 |
| D-dimer(mg/L) | 0.16±0.14 | 0.15±0.11 | 0.18±0.18 | 0.09570173502886441 | 0.15±0.11 | 0.15±0.13 | 0.16±0.06 | 0.7864105786049927 |
| EA-IgA(U/ml) | 2.08±0.61 | 2.10±0.41 | 2.05±0.87 | 0.48967231460792116 | 1.97±0.46 | 2.03±0.32 | 1.85±0.63 | 0.16020678482561904 |
| VCA-IgA(U/ml) | 1.67±0.73 | 1.73±0.80 | 1.56±0.58 | 0.09050154185943783 | 1.72±0.57 | 1.63±0.44 | 1.89±0.73 | 0.10837441846301993 |
| Rta-igG(U/ml) | 0.59±0.14 | 0.59±0.12 | 0.59±0.18 | 0.9150605902071183 | 0.61±0.13 | 0.62±0.11 | 0.59±0.16 | 0.3761998995526584 |
| Ki67(%) | 14.86±24.24 | 13.30±23.70 | 17.66±25.09 | 0.20054154741340727 | 13.33±25.89 | 17.06±27.76 | 6.27±20.79 | 0.1432019739709716 |
| Gender |  |  |  | 0.03019516268662048 |  |  |  | 1.0 |
| Male | 149(67.42) | 88(61.97) | 61(77.22) |  | 41(74.55) | 27(75.00) | 14(73.68) |  |
| Female | 72(32.58) | 54(38.03) | 18(22.78) |  | 14(25.45) | 9(25.00) | 5(26.32) |  |
| Neck_pain |  |  |  | 1.0 |  |  |  | 1.0 |
| Negative | 217(98.19) | 139(97.89) | 78(98.73) |  | 54(98.18) | 35(97.22) | 19(100.00) |  |
| Positive | 4(1.81) | 3(2.11) | 1(1.27) |  | 1(1.82) | 1(2.78) |  |  |
| Headache |  |  |  | 0.10848978687993616 |  |  |  | 1.0 |
| Negative | 179(81.00) | 120(84.51) | 59(74.68) |  | 47(85.45) | 31(86.11) | 16(84.21) |  |
| Positive | 42(19.00) | 22(15.49) | 20(25.32) |  | 8(14.55) | 5(13.89) | 3(15.79) |  |
| Stuffy_nose |  |  |  | 0.8618508281343017 |  |  |  | 0.553674677711491 |
| Negative | 179(81.00) | 116(81.69) | 63(79.75) |  | 47(85.45) | 32(88.89) | 15(78.95) |  |
| Positive | 42(19.00) | 26(18.31) | 16(20.25) |  | 8(14.55) | 4(11.11) | 4(21.05) |  |
| Blood_in_nose |  |  |  | 1.0 |  |  |  | 0.10692975937299701 |
| Negative | 166(75.11) | 107(75.35) | 59(74.68) |  | 38(69.09) | 28(77.78) | 10(52.63) |  |
| Positive | 55(24.89) | 35(24.65) | 20(25.32) |  | 17(30.91) | 8(22.22) | 9(47.37) |  |
| Epistaxis |  |  |  | 0.17466927709826957 |  |  |  | 1.0 |
| Negative | 204(92.31) | 128(90.14) | 76(96.20) |  | 54(98.18) | 35(97.22) | 19(100.00) |  |
| Positive | 17(7.69) | 14(9.86) | 3(3.80) |  | 1(1.82) | 1(2.78) |  |  |
| Vision_loss |  |  |  | 0.7656395940841274 |  |  |  | 1.0 |
| Negative | 220(99.55) | 142(100.00) | 78(98.73) |  | 55(100.00) | 36(100.00) | 19(100.00) |  |
| Positive | 1(0.45) |  | 1(1.27) |  |  |  |  |  |
| Diplopia |  |  |  | 0.5011522898264027 |  |  |  | 1.0 |
| Negative | 216(97.74) | 140(98.59) | 76(96.20) |  | 53(96.36) | 35(97.22) | 18(94.74) |  |
| Positive | 5(2.26) | 2(1.41) | 3(3.80) |  | 2(3.64) | 1(2.78) | 1(5.26) |  |
| Hearing_loss |  |  |  | 0.6778507823725155 |  |  |  | 0.553674677711491 |
| Negative | 197(89.14) | 128(90.14) | 69(87.34) |  | 47(85.45) | 32(88.89) | 15(78.95) |  |
| Positive | 24(10.86) | 14(9.86) | 10(12.66) |  | 8(14.55) | 4(11.11) | 4(21.05) |  |
| Choking |  |  |  | 1.0 |  |  |  | 1.0 |
| Negative | 221(100.00) | 142(100.00) | 79(100.00) |  | 55(100.00) | 36(100.00) | 19(100.00) |  |
| Dysphagia |  |  |  | 0.7656395940841274 |  |  |  | 1.0 |
| Negative | 220(99.55) | 142(100.00) | 78(98.73) |  | 55(100.00) | 36(100.00) | 19(100.00) |  |
| Positive | 1(0.45) |  | 1(1.27) |  |  |  |  |  |
| Back_pain |  |  |  | 1.0 |  |  |  | 1.0 |
| Negative | 221(100.00) | 142(100.00) | 79(100.00) |  | 55(100.00) | 36(100.00) | 19(100.00) |  |
| Limb_pain |  |  |  | 1.0 |  |  |  | 1.0 |
| Negative | 221(100.00) | 142(100.00) | 79(100.00) |  | 55(100.00) | 36(100.00) | 19(100.00) |  |
| Hoarseness |  |  |  | 1.0 |  |  |  | 1.0 |
| Negative | 219(99.10) | 141(99.30) | 78(98.73) |  | 55(100.00) | 36(100.00) | 19(100.00) |  |
| Positive | 2(0.90) | 1(0.70) | 1(1.27) |  |  |  |  |  |
| Facial_numbness |  |  |  | 0.048217269130944956 |  |  |  | 0.22033689174462687 |
| Negative | 211(95.48) | 139(97.89) | 72(91.14) |  | 53(96.36) | 36(100.00) | 17(89.47) |  |
| Positive | 10(4.52) | 3(2.11) | 7(8.86) |  | 2(3.64) |  | 2(10.53) |  |
| Sore_throat |  |  |  | 1.0 |  |  |  | 1.0 |
| Negative | 219(99.10) | 141(99.30) | 78(98.73) |  | 55(100.00) | 36(100.00) | 19(100.00) |  |
| Positive | 2(0.90) | 1(0.70) | 1(1.27) |  |  |  |  |  |
| Other_complaints |  |  |  | 0.33485762822507315 |  |  |  | 1.0 |
| Negative | 205(92.76) | 134(94.37) | 71(89.87) |  | 54(98.18) | 35(97.22) | 19(100.00) |  |
| Positive | 16(7.24) | 8(5.63) | 8(10.13) |  | 1(1.82) | 1(2.78) |  |  |
| Family_history |  |  |  | 1.0 |  |  |  | 1.0 |
| Negative | 217(98.19) | 139(97.89) | 78(98.73) |  | 48(87.27) | 31(86.11) | 17(89.47) |  |
| Positive | 4(1.81) | 3(2.11) | 1(1.27) |  | 7(12.73) | 5(13.89) | 2(10.53) |  |
| Histology |  |  |  | <0.001 |  |  |  | 0.1462293298790989 |
| Non-keratinizing squamous cell carcinoma | 127(57.47) | 87(61.27) | 40(50.63) |  | 29(52.73) | 21(58.33) | 8(42.11) |  |
| Keratinizing squamous cell carcinoma | 35(15.84) | 11(7.75) | 24(30.38) |  | 12(21.82) | 5(13.89) | 7(36.84) |  |
| Basaloid squamous cell carcinoma | 59(26.70) | 44(30.99) | 15(18.99) |  | 14(25.45) | 10(27.78) | 4(21.05) |  |
| T_staging |  |  |  | 0.06640687809679532 |  |  |  | 0.05327538274201606 |
| 1 | 6(2.71) | 3(2.11) | 3(3.80) |  |  |  |  |  |
| 2 | 29(13.12) | 21(14.79) | 8(10.13) |  | 6(10.91) | 6(16.67) |  |  |
| 3 | 98(44.34) | 70(49.30) | 28(35.44) |  | 32(58.18) | 22(61.11) | 10(52.63) |  |
| 4 | 88(39.82) | 48(33.80) | 40(50.63) |  | 17(30.91) | 8(22.22) | 9(47.37) |  |
| N_staging |  |  |  | 0.002248891544205016 |  |  |  | 0.04900006993189538 |
| 0 | 6(2.71) | 3(2.11) | 3(3.80) |  |  |  |  |  |
| 1 | 56(25.34) | 26(18.31) | 30(37.97) |  | 8(14.55) | 4(11.11) | 4(21.05) |  |
| 2 | 82(37.10) | 53(37.32) | 29(36.71) |  | 23(41.82) | 12(33.33) | 11(57.89) |  |
| 3 | 77(34.84) | 60(42.25) | 17(21.52) |  | 24(43.64) | 20(55.56) | 4(21.05) |  |
| TNM Staging |  |  |  | 0.13439420012357275 |  |  |  | 0.4672666160260297 |
| III | 117(52.94) | 81(57.04) | 36(45.57) |  | 34(61.82) | 24(66.67) | 10(52.63) |  |
| IVA | 104(47.06) | 61(42.96) | 43(54.43) |  | 21(38.18) | 12(33.33) | 9(47.37) |  |
| Treatment |  |  |  | 0.06994257004968361 |  |  |  | 0.30300819316258354 |
| Paclitaxel + cisplatin (TP) | 20(9.05) | 11(7.75) | 9(11.39) |  | 1(1.82) |  | 1(5.26) |  |
| Fluorouracil + cisplatin (PF) | 1(0.45) |  | 1(1.27) |  |  |  |  |  |
| Gemcitabine + cisplatin (GP) | 53(23.98) | 36(25.35) | 17(21.52) |  | 17(30.91) | 11(30.56) | 6(31.58) |  |
| Docetaxel + cisplatin(DP) | 98(44.34) | 57(40.14) | 41(51.90) |  | 21(38.18) | 11(30.56) | 10(52.63) |  |
| Paclitaxel + cisplatin + fluorouracil (TPF) | 1(0.45) |  | 1(1.27) |  |  |  |  |  |
| Sintilimab + GP | 10(4.52) | 10(7.04) |  |  | 4(7.27) | 4(11.11) |  |  |
| Toripalimab + GP |  |  |  |  | 2(3.64) | 2(5.56) |  |  |
| Tislelizumab + GP | 1(0.45) | 1(0.70) |  |  |  |  |  |  |
| Camrelizumab + GP | 1(0.45) | 1(0.70) |  |  |  |  |  |  |
| Nivolumab + GP | 25(11.31) | 20(14.08) | 5(6.33) |  | 7(12.73) | 5(13.89) | 2(10.53) |  |
| Endostatin + DP | 7(3.17) | 3(2.11) | 4(5.06) |  | 2(3.64) | 2(5.56) |  |  |
| Nimotuzumab + TPF | 4(1.81) | 3(2.11) | 1(1.27) |  | 1(1.82) | 1(2.78) |  |  |
| Category |  |  |  | 0.007813401881697988 |  |  |  | 0.0778881363187681 |
| Immunotherapy + induction chemotherapy | 37(16.74) | 32(22.54) | 5(6.33) |  | 13(23.64) | 11(30.56) | 2(10.53) |  |
| Targeted therapy | 11(4.98) | 6(4.23) | 5(6.33) |  | 3(5.45) | 3(8.33) |  |  |
| Induction chemotherapy | 173(78.28) | 104(73.24) | 69(87.34) |  | 39(70.91) | 22(61.11) | 17(89.47) |  |
| EGFR |  |  |  | 0.05694863938150966 |  |  |  | 0.8524130078233872 |
| (-) | 139(62.90) | 98(69.01) | 41(51.90) |  | 37(67.27) | 23(63.89) | 14(73.68) |  |
| (+) | 37(16.74) | 19(13.38) | 18(22.78) |  | 6(10.91) | 4(11.11) | 2(10.53) |  |
| (++) | 17(7.69) | 11(7.75) | 6(7.59) |  | 3(5.45) | 2(5.56) | 1(5.26) |  |
| (+++) | 28(12.67) | 14(9.86) | 14(17.72) |  | 9(16.36) | 7(19.44) | 2(10.53) |  |
| P53 |  |  |  | 0.45342404288758775 |  |  |  | 0.5626318012408469 |
| (-) | 209(94.57) | 136(95.77) | 73(92.41) |  | 52(94.55) | 35(97.22) | 17(89.47) |  |
| (+) | 12(5.43) | 6(4.23) | 6(7.59) |  | 3(5.45) | 1(2.78) | 2(10.53) |  |
| EBER |  |  |  | 0.11353493503647785 |  |  |  | 0.5258980191161703 |
| (-) | 137(61.99) | 94(66.20) | 43(54.43) |  | 36(65.45) | 22(61.11) | 14(73.68) |  |
| (+) | 84(38.01) | 48(33.80) | 36(45.57) |  | 19(34.55) | 14(38.89) | 5(26.32) |  |
| P63 |  |  |  | 0.06787143525561935 |  |  |  | 0.8315811875191995 |
| (-) | 168(76.02) | 114(80.28) | 54(68.35) |  | 44(80.00) | 28(77.78) | 16(84.21) |  |
| (+) | 53(23.98) | 28(19.72) | 25(31.65) |  | 11(20.00) | 8(22.22) | 3(15.79) |  |
| P40 |  |  |  | 0.09216775348351473 |  |  |  | 1.0 |
| (-) | 172(77.83) | 116(81.69) | 56(70.89) |  | 40(72.73) | 26(72.22) | 14(73.68) |  |
| (+) | 49(22.17) | 26(18.31) | 23(29.11) |  | 15(27.27) | 10(27.78) | 5(26.32) |  |

| 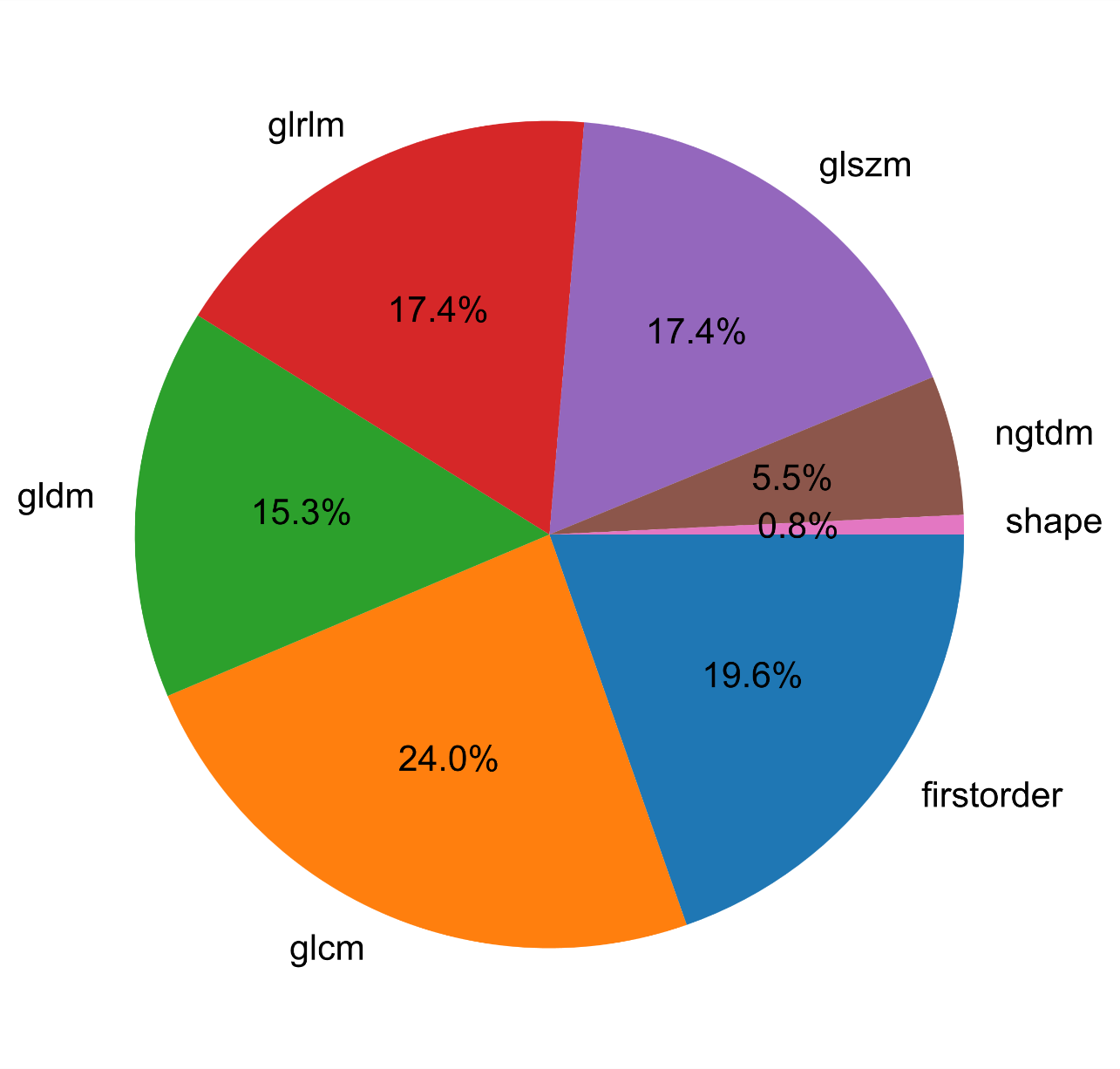  Figure S2: **Ratio of all handcrafted features for each region** |
| --- |

| 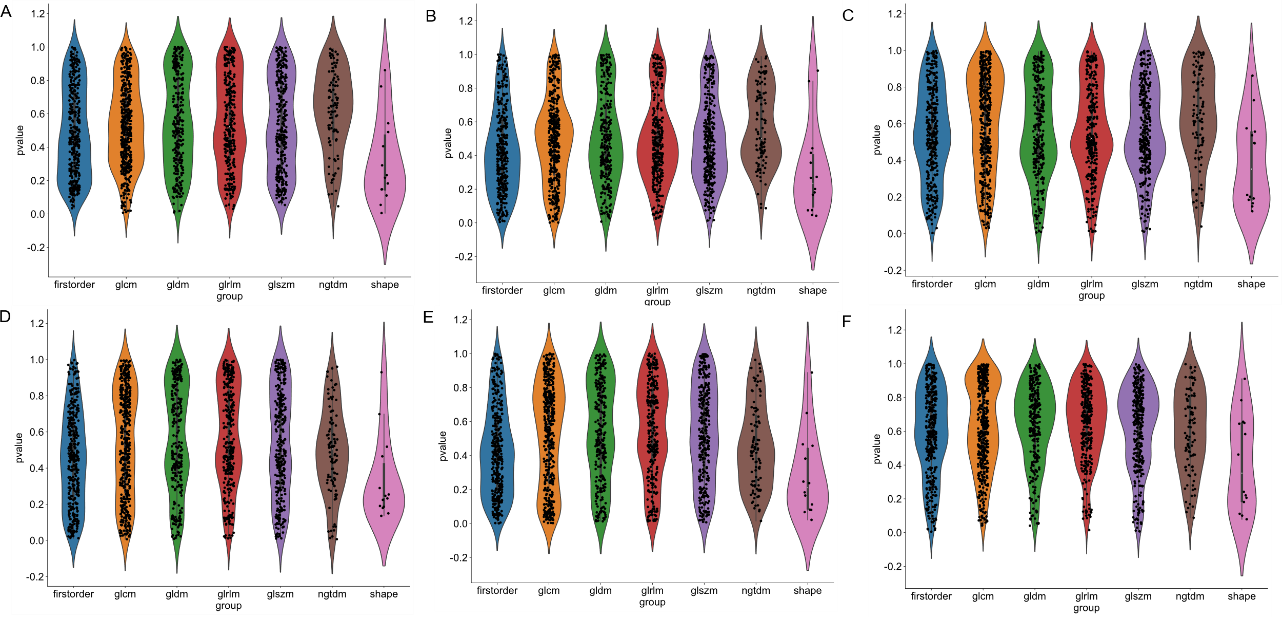  Figure S3: **Statistics of all extracted radiomic features** (Subplot A represents intratumoral region, while sublots B-F represent peritumoral dilation region 1-5 mm, respectively) |
| --- |

Table S2: **Model performances of all intratumoral and peritumoral regions**

| model_name | Accuracy | AUC | 95% CI | Sensitivity | Specificity | PPV | NPV | Precision | Recall | F1 | Threshold | Task | Dilation |
| --- | --- | --- | --- | --- | --- | --- | --- | --- | --- | --- | --- | --- | --- |
| LR | 0.714932 | 0.73489 | 0.6673 - 0.8024 | 0.810127 | 0.666667 | 0.571429 | 0.862385 | 0.571429 | 0.810127 | 0.670157 | 0.341836 | label-train | 0 |
| LR | 0.727273 | 0.641813 | 0.4795 - 0.8041 | 0.526316 | 0.833333 | 0.625 | 0.769231 | 0.625 | 0.526316 | 0.571429 | 0.467496 | label-test | 0 |
| SVM | 0.828054 | 0.849795 | 0.7912 - 0.9084 | 0.772152 | 0.859155 | 0.753086 | 0.871429 | 0.753086 | 0.772152 | 0.7625 | 0.381609 | label-train | 0 |
| SVM | 0.654545 | 0.625731 | 0.4654 - 0.7860 | 0.526316 | 0.742857 | 0.5 | 0.742857 | 0.5 | 0.526316 | 0.51282 | 0.403709 | label-test | 0 |
| KNN | 0.683258 | 0.780175 | 0.7227 - 0.8376 | 0.873418 | 0.577465 | 0.534884 | 0.891304 | 0.534884 | 0.873418 | 0.663462 | 0.4 | label-train | 0 |
| KNN | 0.509091 | 0.511696 | 0.3566 - 0.6668 | 0.578947 | 0.485714 | 0.366667 | 0.68 | 0.366667 | 0.578947 | 0.44898 | 0.4 | label-test | 0 |
| RandomForest | 0.9819 | 0.999198 | 0.9980 - 1.0000 | 0.987342 | 0.978873 | 0.962963 | 0.992857 | 0.962963 | 0.987342 | 0.975 | 0.5 | label-train | 0 |
| RandomForest | 0.563636 | 0.606725 | 0.4508 - 0.7627 | 0.736842 | 0.5 | 0.424242 | 0.772727 | 0.424242 | 0.736842 | 0.538462 | 0.4 | label-test | 0 |
| ExtraTrees | 1 | 1 | 1.0000 - 1.0000 | 1 | 1 | 1 | 1 | 1 | 1 | 1 | 1 | label-train | 0 |
| ExtraTrees | 0.636364 | 0.633772 | 0.4809 - 0.7867 | 0.684211 | 0.628571 | 0.481481 | 0.785714 | 0.481481 | 0.684211 | 0.565217 | 0.4 | label-test | 0 |
| XGBoost | 0.9819 | 0.995275 | 0.9890 - 1.0000 | 0.962025 | 0.992958 | 0.987013 | 0.979167 | 0.987013 | 0.962025 | 0.974359 | 0.397832 | label-train | 0 |
| XGBoost | 0.690909 | 0.590643 | 0.4274 - 0.7539 | 0.473684 | 0.828571 | 0.5625 | 0.74359 | 0.5625 | 0.473684 | 0.514286 | 0.480967 | label-test | 0 |
| LightGBM | 0.823529 | 0.894411 | 0.8514 - 0.9374 | 0.911392 | 0.774648 | 0.692308 | 0.940171 | 0.692308 | 0.911392 | 0.786885 | 0.37014 | label-train | 0 |
| LightGBM | 0.672727 | 0.612573 | 0.4429 - 0.7823 | 0.473684 | 0.777778 | 0.529412 | 0.736842 | 0.529412 | 0.473684 | 0.5 | 0.439753 | label-test | 0 |
| MLP | 0.751131 | 0.772063 | 0.7081 - 0.8360 | 0.822785 | 0.711268 | 0.613208 | 0.878261 | 0.613208 | 0.822785 | 0.702703 | 0.35501 | label-train | 0 |
| MLP | 0.745455 | 0.654971 | 0.4947 - 0.8153 | 0.473684 | 0.888889 | 0.692308 | 0.761905 | 0.692308 | 0.473684 | 0.5625 | 0.498835 | label-test | 0 |
| LR | 0.773756 | 0.7775 | 0.7134 - 0.8416 | 0.594937 | 0.873239 | 0.723077 | 0.794872 | 0.723077 | 0.594937 | 0.652778 | 0.460058 | label-train | 1 |
| LR | 0.581818 | 0.574561 | 0.4169 - 0.7323 | 0.789474 | 0.485714 | 0.441176 | 0.809524 | 0.441176 | 0.789474 | 0.566038 | 0.302182 | label-test | 1 |
| SVM | 0.841629 | 0.93448 | 0.9028 - 0.9662 | 0.898734 | 0.809859 | 0.72449 | 0.934959 | 0.72449 | 0.898734 | 0.80226 | 0.307098 | label-train | 1 |
| SVM | 0.636364 | 0.602339 | 0.4462 - 0.7584 | 0.526316 | 0.694444 | 0.47619 | 0.735294 | 0.47619 | 0.526316 | 0.5 | 0.396639 | label-test | 1 |
| KNN | 0.773756 | 0.797602 | 0.7398 - 0.8554 | 0.582278 | 0.880282 | 0.730159 | 0.791139 | 0.730159 | 0.582278 | 0.647887 | 0.6 | label-train | 1 |
| KNN | 0.436364 | 0.552632 | 0.4025 - 0.7027 | 0.947368 | 0.181818 | 0.375 | 0.857143 | 0.375 | 0.947368 | 0.537313 | 0.2 | label-test | 1 |
| RandomForest | 0.977376 | 0.998485 | 0.9966 - 1.0000 | 0.974684 | 0.978873 | 0.9625 | 0.985816 | 0.9625 | 0.974684 | 0.968553 | 0.5 | label-train | 1 |
| RandomForest | 0.690909 | 0.494883 | 0.3285 - 0.6613 | 0.105263 | 1 | 1 | 0.679245 | 1 | 0.105263 | 0.190476 | 0.9 | label-test | 1 |
| ExtraTrees | 1 | 1 | 1.0000 - 1.0000 | 1 | 1 | 1 | 1 | 1 | 1 | 1 | 1 | label-train | 1 |
| ExtraTrees | 0.472727 | 0.527778 | 0.3757 - 0.6799 | 0.947368 | 0.235294 | 0.391304 | 0.888889 | 0.391304 | 0.947368 | 0.553846 | 0.3 | label-test | 1 |
| XGBoost | 1 | 1 | 1.0000 - 1.0000 | 1 | 1 | 1 | 1 | 1 | 1 | 1 | 0.487031 | label-train | 1 |
| XGBoost | 0.618182 | 0.608187 | 0.4532 - 0.7632 | 0.631579 | 0.611111 | 0.461538 | 0.758621 | 0.461538 | 0.631579 | 0.533333 | 0.335755 | label-test | 1 |
| LightGBM | 0.886878 | 0.965056 | 0.9457 - 0.9844 | 0.898734 | 0.880282 | 0.806818 | 0.93985 | 0.806818 | 0.898734 | 0.850299 | 0.39245 | label-train | 1 |
| LightGBM | 0.527273 | 0.548246 | 0.3906 - 0.7059 | 0.789474 | 0.388889 | 0.405405 | 0.777778 | 0.405405 | 0.789474 | 0.535714 | 0.306685 | label-test | 1 |
| MLP | 0.800905 | 0.88046 | 0.8362 - 0.9247 | 0.835443 | 0.78169 | 0.680412 | 0.895161 | 0.680412 | 0.835443 | 0.75 | 0.324501 | label-train | 1 |
| MLP | 0.6 | 0.567251 | 0.4116 - 0.7229 | 0.684211 | 0.571429 | 0.448276 | 0.769231 | 0.448276 | 0.684211 | 0.541667 | 0.311286 | label-test | 1 |
| LR | 0.728507 | 0.727937 | 0.6561 - 0.7997 | 0.56962 | 0.816901 | 0.633803 | 0.773333 | 0.633803 | 0.56962 | 0.6 | 0.424903 | label-train | 2 |
| LR | 0.727273 | 0.666667 | 0.5178 - 0.8155 | 0.789474 | 0.714286 | 0.576923 | 0.862069 | 0.576923 | 0.789474 | 0.666667 | 0.3591 | label-test | 2 |
| SVM | 0.791855 | 0.839544 | 0.7798 - 0.8993 | 0.848101 | 0.760563 | 0.663366 | 0.9 | 0.663366 | 0.848101 | 0.744444 | 0.328574 | label-train | 2 |
| SVM | 0.581818 | 0.597953 | 0.4369 - 0.7590 | 0.842105 | 0.444444 | 0.444444 | 0.842105 | 0.444444 | 0.842105 | 0.581818 | 0.332548 | label-test | 2 |
| KNN | 0.769231 | 0.79778 | 0.7416 - 0.8540 | 0.468354 | 0.943262 | 0.804348 | 0.76 | 0.804348 | 0.468354 | 0.592 | 0.6 | label-train | 2 |
| KNN | 0.527273 | 0.543129 | 0.3863 - 0.6999 | 0.631579 | 0.472222 | 0.387097 | 0.708333 | 0.387097 | 0.631579 | 0.48 | 0.4 | label-test | 2 |
| RandomForest | 0.977376 | 0.996835 | 0.9926 - 1.0000 | 1 | 0.964789 | 0.940476 | 1 | 0.940476 | 1 | 0.969325 | 0.4 | label-train | 2 |
| RandomForest | 0.618182 | 0.624269 | 0.4721 - 0.7765 | 0.736842 | 0.555556 | 0.466667 | 0.8 | 0.466667 | 0.736842 | 0.571429 | 0.4 | label-test | 2 |
| ExtraTrees | 1 | 1 | 1.0000 - 1.0000 | 1 | 1 | 1 | 1 | 1 | 1 | 1 | 1 | label-train | 2 |
| ExtraTrees | 0.654545 | 0.693713 | 0.5501 - 0.8373 | 0.684211 | 0.657143 | 0.5 | 0.793103 | 0.5 | 0.684211 | 0.577778 | 0.4 | label-test | 2 |
| XGBoost | 0.995475 | 0.998752 | 0.9962 - 1.0000 | 1 | 0.992958 | 0.9875 | 1 | 0.9875 | 1 | 0.993711 | 0.520984 | label-train | 2 |
| XGBoost | 0.727273 | 0.682749 | 0.5313 - 0.8342 | 0.842105 | 0.666667 | 0.571429 | 0.888889 | 0.571429 | 0.842105 | 0.680851 | 0.332525 | label-test | 2 |
| LightGBM | 0.841629 | 0.921778 | 0.8884 - 0.9551 | 0.886076 | 0.816901 | 0.729167 | 0.928 | 0.729167 | 0.886076 | 0.8 | 0.371688 | label-train | 2 |
| LightGBM | 0.618182 | 0.613304 | 0.4492 - 0.7774 | 0.684211 | 0.6 | 0.464286 | 0.777778 | 0.464286 | 0.684211 | 0.553191 | 0.359639 | label-test | 2 |
| MLP | 0.714932 | 0.747281 | 0.6789 - 0.8157 | 0.670886 | 0.739437 | 0.588889 | 0.801527 | 0.588889 | 0.670886 | 0.627219 | 0.364834 | label-train | 2 |
| MLP | 0.672727 | 0.659357 | 0.5113 - 0.8074 | 0.789474 | 0.611111 | 0.517241 | 0.846154 | 0.517241 | 0.789474 | 0.625 | 0.346046 | label-test | 2 |
| LR | 0.696833 | 0.735158 | 0.6644 - 0.8059 | 0.64557 | 0.725352 | 0.566667 | 0.78626 | 0.566667 | 0.64557 | 0.60355 | 0.358517 | label-train | 3 |
| LR | 0.490909 | 0.457602 | 0.3028 - 0.6124 | 0.736842 | 0.371429 | 0.378378 | 0.722222 | 0.378378 | 0.736842 | 0.5 | 0.222236 | label-test | 3 |
| SVM | 0.832579 | 0.896862 | 0.8535 - 0.9402 | 0.772152 | 0.866197 | 0.7625 | 0.87234 | 0.7625 | 0.772152 | 0.767296 | 0.367548 | label-train | 3 |
| SVM | 0.563636 | 0.491228 | 0.3262 - 0.6563 | 0.684211 | 0.514286 | 0.419355 | 0.75 | 0.419355 | 0.684211 | 0.52 | 0.329819 | label-test | 3 |
| KNN | 0.728507 | 0.828668 | 0.7771 - 0.8803 | 0.797468 | 0.690141 | 0.588785 | 0.859649 | 0.588785 | 0.797468 | 0.677419 | 0.4 | label-train | 3 |
| KNN | 0.436364 | 0.509503 | 0.3662 - 0.6528 | 0.947368 | 0.193548 | 0.375 | 0.857143 | 0.375 | 0.947368 | 0.537313 | 0.2 | label-test | 3 |
| RandomForest | 0.9819 | 0.998217 | 0.9961 - 1.0000 | 0.974684 | 0.985915 | 0.974684 | 0.985915 | 0.974684 | 0.974684 | 0.974684 | 0.5 | label-train | 3 |
| RandomForest | 0.636364 | 0.554094 | 0.3920 - 0.7162 | 0.368421 | 0.777778 | 0.466667 | 0.7 | 0.466667 | 0.368421 | 0.411765 | 0.5 | label-test | 3 |
| ExtraTrees | 1 | 1 | 1.0000 - 1.0000 | 1 | 1 | 1 | 1 | 1 | 1 | 1 | 1 | label-train | 3 |
| ExtraTrees | 0.581818 | 0.54386 | 0.3801 - 0.7076 | 0.631579 | 0.571429 | 0.428571 | 0.740741 | 0.428571 | 0.631579 | 0.510638 | 0.4 | label-test | 3 |
| XGBoost | 1 | 1 | 1.0000 - 1.0000 | 1 | 1 | 1 | 1 | 1 | 1 | 1 | 0.407683 | label-train | 3 |
| XGBoost | 0.618182 | 0.52924 | 0.3563 - 0.7022 | 0.473684 | 0.714286 | 0.45 | 0.714286 | 0.45 | 0.473684 | 0.461538 | 0.428228 | label-test | 3 |
| LightGBM | 0.877828 | 0.924006 | 0.8888 - 0.9592 | 0.848101 | 0.894366 | 0.817073 | 0.913669 | 0.817073 | 0.848101 | 0.832298 | 0.40336 | label-train | 3 |
| LightGBM | 0.654545 | 0.519006 | 0.3504 - 0.6876 | 0.473684 | 0.771429 | 0.5 | 0.72973 | 0.5 | 0.473684 | 0.486486 | 0.379146 | label-test | 3 |
| MLP | 0.769231 | 0.802282 | 0.7419 - 0.8626 | 0.632911 | 0.84507 | 0.694444 | 0.805369 | 0.694444 | 0.632911 | 0.662252 | 0.418984 | label-train | 3 |
| MLP | 0.490909 | 0.453216 | 0.2957 - 0.6107 | 0.736842 | 0.371429 | 0.378378 | 0.722222 | 0.378378 | 0.736842 | 0.5 | 0.288272 | label-test | 3 |
| LR | 0.728507 | 0.819665 | 0.7654 - 0.8740 | 0.835443 | 0.669014 | 0.584071 | 0.87963 | 0.584071 | 0.835443 | 0.6875 | 0.294233 | label-train | 4 |
| LR | 0.381818 | 0.337719 | 0.1921 - 0.4834 | 0.894737 | 0.114286 | 0.346939 | 0.666667 | 0.346939 | 0.894737 | 0.5 | 0.0923542 | label-test | 4 |
| SVM | 0.877828 | 0.932385 | 0.8978 - 0.9670 | 0.860759 | 0.887324 | 0.809524 | 0.919708 | 0.809524 | 0.860759 | 0.834356 | 0.357396 | label-train | 4 |
| SVM | 0.345455 | 0.30848 | 0.1600 - 0.4569 | 1 | 0 | 0.345455 | 0 | 0.345455 | 1 | 0.513514 | 0.115961 | label-test | 4 |
| KNN | 0.769231 | 0.824122 | 0.7723 - 0.8759 | 0.810127 | 0.746479 | 0.64 | 0.876033 | 0.64 | 0.810127 | 0.715084 | 0.4 | label-train | 4 |
| KNN | 0.454545 | 0.498538 | 0.3507 - 0.6463 | 0.894737 | 0.228571 | 0.377778 | 0.8 | 0.377778 | 0.894737 | 0.53125 | 0.2 | label-test | 4 |
| RandomForest | 0.986425 | 0.99893 | 0.9974 - 1.0000 | 1 | 0.978873 | 0.963415 | 1 | 0.963415 | 1 | 0.981366 | 0.5 | label-train | 4 |
| RandomForest | 0.363636 | 0.326023 | 0.1780 - 0.4741 | 1 | 0.0285714 | 0.351852 | 0.999999 | 0.351852 | 1 | 0.520548 | 0.1 | label-test | 4 |
| ExtraTrees | 1 | 1 | 1.0000 - 1.0000 | 1 | 1 | 1 | 1 | 1 | 1 | 1 | 1 | label-train | 4 |
| ExtraTrees | 0.418182 | 0.431287 | 0.2764 - 0.5862 | 0.894737 | 0.171429 | 0.361702 | 0.75 | 0.361702 | 0.894737 | 0.515152 | 0.2 | label-test | 4 |
| XGBoost | 1 | 1 | 1.0000 - 1.0000 | 1 | 1 | 1 | 1 | 1 | 1 | 1 | 0.522662 | label-train | 4 |
| XGBoost | 0.363636 | 0.266082 | 0.1288 - 0.4034 | 1 | 0.0285714 | 0.351852 | 0.999999 | 0.351852 | 1 | 0.520548 | 0.0557536 | label-test | 4 |
| LightGBM | 0.882353 | 0.953601 | 0.9296 - 0.9776 | 0.860759 | 0.894366 | 0.819277 | 0.92029 | 0.819277 | 0.860759 | 0.839506 | 0.425553 | label-train | 4 |
| LightGBM | 0.490909 | 0.42617 | 0.2696 - 0.5827 | 0.684211 | 0.4 | 0.371429 | 0.7 | 0.371429 | 0.684211 | 0.481481 | 0.343386 | label-test | 4 |
| MLP | 0.791855 | 0.879569 | 0.8354 - 0.9238 | 0.886076 | 0.739437 | 0.654206 | 0.921053 | 0.654206 | 0.886076 | 0.752688 | 0.342301 | label-train | 4 |
| MLP | 0.418182 | 0.365497 | 0.2181 - 0.5129 | 1 | 0.114286 | 0.372549 | 1 | 0.372549 | 1 | 0.542857 | 0.0845516 | label-test | 4 |
| LR | 0.660633 | 0.701195 | 0.6285 - 0.7739 | 0.658228 | 0.661972 | 0.52 | 0.776859 | 0.52 | 0.658228 | 0.581006 | 0.350204 | label-train | 5 |
| LR | 0.4 | 0.44883 | 0.2910 - 0.6066 | 1 | 0.0857143 | 0.365385 | 1 | 0.365385 | 1 | 0.535211 | 0.10772 | label-test | 5 |
| SVM | 0.782805 | 0.877162 | 0.8285 - 0.9259 | 0.949367 | 0.690141 | 0.630252 | 0.960784 | 0.630252 | 0.949367 | 0.757576 | 0.291207 | label-train | 5 |
| SVM | 0.363636 | 0.399123 | 0.2389 - 0.5593 | 1 | 0.0285714 | 0.351852 | 0.999999 | 0.351852 | 1 | 0.520548 | 0.243608 | label-test | 5 |
| KNN | 0.701357 | 0.791897 | 0.7346 - 0.8492 | 0.772152 | 0.661972 | 0.559633 | 0.839286 | 0.559633 | 0.772152 | 0.648936 | 0.4 | label-train | 5 |
| KNN | 0.545455 | 0.490497 | 0.3310 - 0.6500 | 0.473684 | 0.583333 | 0.375 | 0.677419 | 0.375 | 0.473684 | 0.418605 | 0.4 | label-test | 5 |
| RandomForest | 0.986425 | 0.999287 | 0.9982 - 1.0000 | 0.987342 | 0.985915 | 0.975 | 0.992908 | 0.975 | 0.987342 | 0.981132 | 0.5 | label-train | 5 |
| RandomForest | 0.509091 | 0.491228 | 0.3261 - 0.6563 | 0.578947 | 0.485714 | 0.366667 | 0.68 | 0.366667 | 0.578947 | 0.44898 | 0.4 | label-test | 5 |
| ExtraTrees | 1 | 1 | 1.0000 - 1.0000 | 1 | 1 | 1 | 1 | 1 | 1 | 1 | 1 | label-train | 5 |
| ExtraTrees | 0.345455 | 0.445906 | 0.2815 - 0.6103 | 1 | 0 | 0.345455 | 0 | 0.345455 | 1 | 0.513514 | 0 | label-test | 5 |
| XGBoost | 1 | 1 | 1.0000 - 1.0000 | 1 | 1 | 1 | 1 | 1 | 1 | 1 | 0.461483 | label-train | 5 |
| XGBoost | 0.6 | 0.447368 | 0.2816 - 0.6131 | 0.315789 | 0.771429 | 0.4 | 0.675 | 0.4 | 0.315789 | 0.352941 | 0.612275 | label-test | 5 |
| LightGBM | 0.791855 | 0.878231 | 0.8336 - 0.9229 | 0.911392 | 0.725352 | 0.648649 | 0.936364 | 0.648649 | 0.911392 | 0.757895 | 0.339665 | label-train | 5 |
| LightGBM | 0.472727 | 0.519006 | 0.3637 - 0.6743 | 1 | 0.2 | 0.395833 | 1 | 0.395833 | 1 | 0.567164 | 0.206057 | label-test | 5 |
| MLP | 0.683258 | 0.753789 | 0.6877 - 0.8199 | 0.78481 | 0.626761 | 0.53913 | 0.839623 | 0.53913 | 0.78481 | 0.639175 | 0.316495 | label-train | 5 |
| MLP | 0.418182 | 0.434211 | 0.2742 - 0.5942 | 0.894737 | 0.171429 | 0.361702 | 0.75 | 0.361702 | 0.894737 | 0.515152 | 0.207722 | label-test | 5 |

| 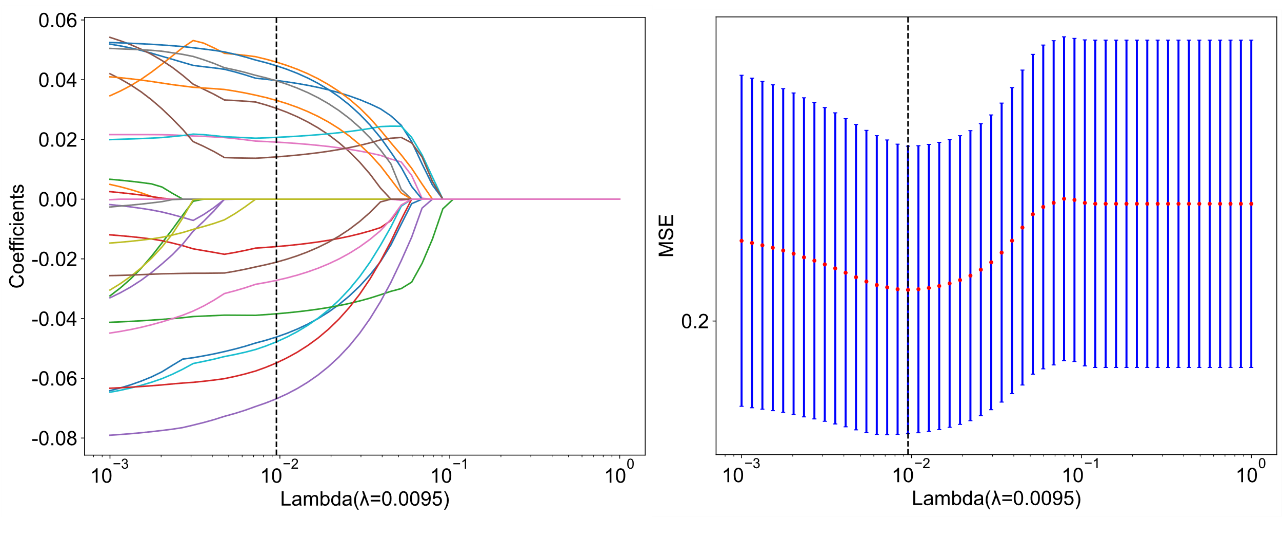  Figure S4: **LASSO coefficients(left) and MSE(right) of combined radiomics signature(right)** |
| --- |

| 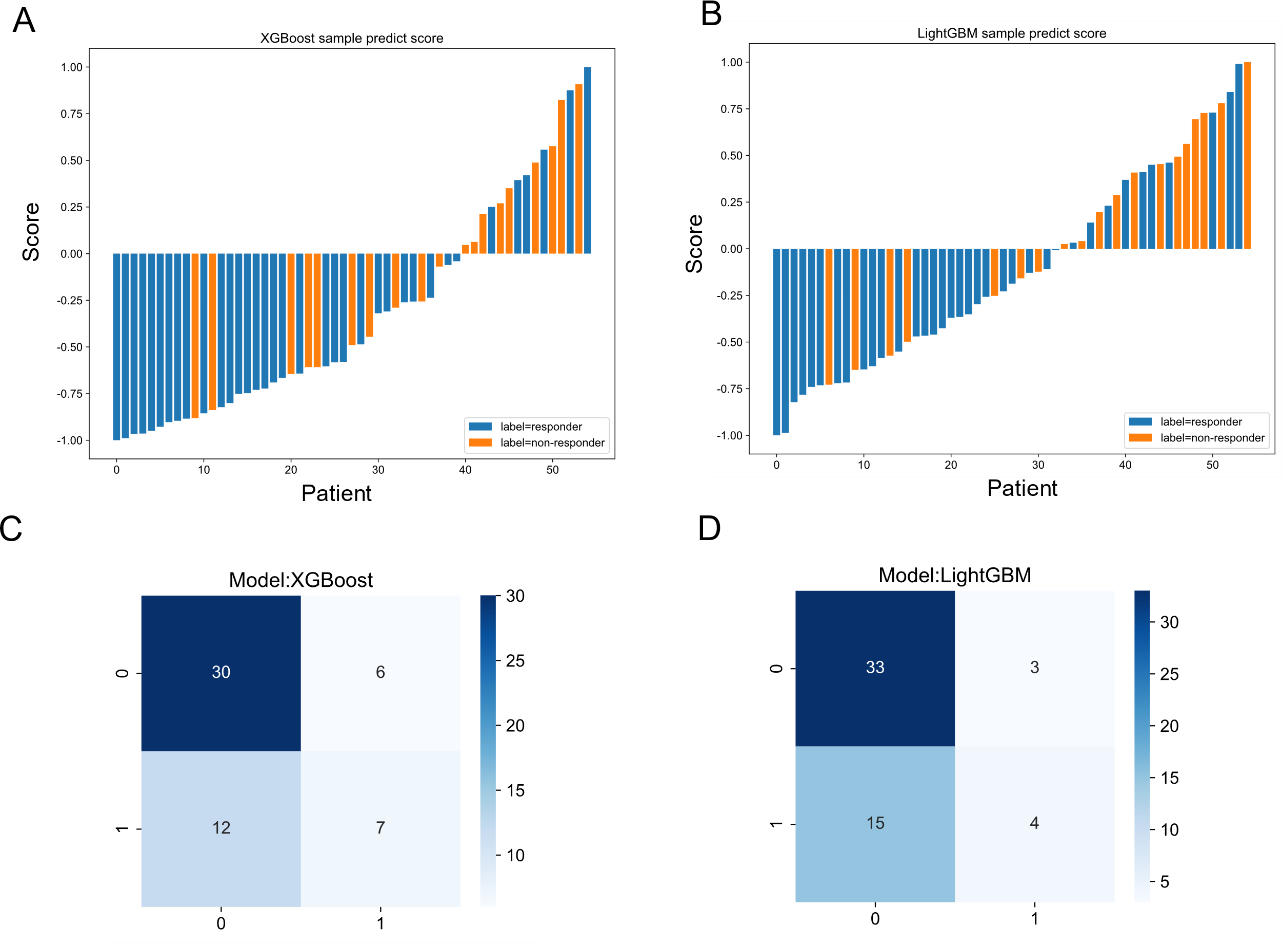  Figure S5: **Prediction score histogram(A,B) and confusion matrix(C,D) of 2 best performing models on testing cohort** |
| --- |

Table S3: **Model performances of clinic features**

| model_name | Accuracy | AUC | 95% CI | Sensitivity | Specificity | PPV | NPV | Precision | Recall | F1 | Threshold | Task |
| --- | --- | --- | --- | --- | --- | --- | --- | --- | --- | --- | --- | --- |
| LR | 0.656109 | 0.724015 | 0.6544 - 0.7936 | 0.78481 | 0.584507 | 0.512397 | 0.83 | 0.512397 | 0.78481 | 0.62 | 0.306207 | label-train |
| LR | 0.690909 | 0.71345 | 0.5697 - 0.8572 | 0.842105 | 0.611111 | 0.533333 | 0.88 | 0.533333 | 0.842105 | 0.653061 | 0.261922 | label-test |
| SVM | 0.669683 | 0.63875 | 0.5592 - 0.7183 | 0.708861 | 0.647887 | 0.528302 | 0.8 | 0.528302 | 0.708861 | 0.605405 | 0.337913 | label-train |
| SVM | 0.709091 | 0.440058 | 0.2657 - 0.6144 | 0.157895 | 1 | 1 | 0.692308 | 1 | 0.157895 | 0.272727 | 0.565928 | label-test |
| KNN | 0.773756 | 0.881441 | 0.8407 - 0.9222 | 0.949367 | 0.676056 | 0.619835 | 0.96 | 0.619835 | 0.949367 | 0.75 | 0.4 | label-train |
| KNN | 0.581818 | 0.660819 | 0.5157 - 0.8060 | 0.842105 | 0.484848 | 0.444444 | 0.842105 | 0.444444 | 0.842105 | 0.581818 | 0.2 | label-test |
| RandomForest | 0.9819 | 0.998752 | 0.9971 - 1.0000 | 0.974684 | 0.985915 | 0.974684 | 0.985915 | 0.974684 | 0.974684 | 0.974684 | 0.5 | label-train |
| RandomForest | 0.690909 | 0.72076 | 0.5838 - 0.8577 | 0.736842 | 0.666667 | 0.538462 | 0.827586 | 0.538462 | 0.736842 | 0.622222 | 0.3 | label-test |
| ExtraTrees | 1 | 1 | 1.0000 - 1.0000 | 1 | 1 | 1 | 1 | 1 | 1 | 1 | 1 | label-train |
| ExtraTrees | 0.763636 | 0.730994 | 0.5806 - 0.8814 | 0.684211 | 0.805556 | 0.65 | 0.828571 | 0.65 | 0.684211 | 0.666667 | 0.5 | label-test |
| XGBoost | 0.968326 | 0.988857 | 0.9768 - 1.0000 | 0.936709 | 0.985915 | 0.973684 | 0.965517 | 0.973684 | 0.936709 | 0.954839 | 0.525636 | label-train |
| XGBoost | 0.745455 | 0.739766 | 0.6070 - 0.8726 | 0.789474 | 0.742857 | 0.6 | 0.866667 | 0.6 | 0.789474 | 0.681818 | 0.391791 | label-test |
| LightGBM | 0.79638 | 0.902968 | 0.8640 - 0.9419 | 0.911392 | 0.732394 | 0.654545 | 0.936937 | 0.654545 | 0.911392 | 0.761905 | 0.394761 | label-train |
| LightGBM | 0.690909 | 0.772661 | 0.6471 - 0.8983 | 0.894737 | 0.583333 | 0.53125 | 0.913043 | 0.53125 | 0.894737 | 0.666667 | 0.294538 | label-test |
| MLP | 0.556561 | 0.65493 | 0.6168 - 0.6931 | 1 | 1 | 0.446328 | 1 | 0.446328 | 1 | 0.617187 | 0.405199 | label-train |
| MLP | 0.563636 | 0.666667 | 0.5886 - 0.7448 | 1 | 1 | 0.44186 | 1 | 0.44186 | 1 | 0.612903 | 0.405199 | label-test |

| 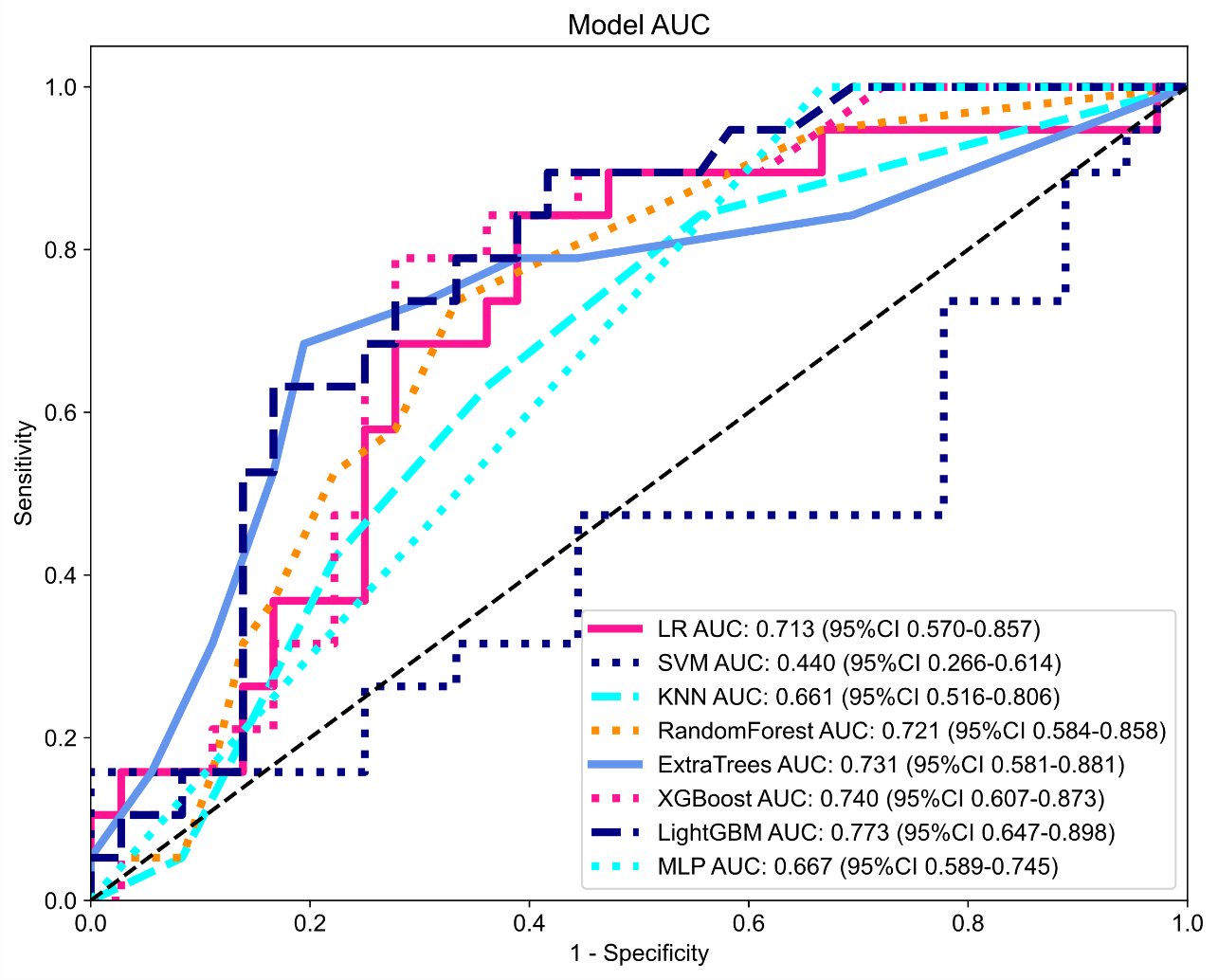  Figure S6: **ROC analysis of different models on clinical signature in testing cohort** |
| --- |

Table S4: **Comparisons of different models in delong test**

| Nomogram Vs Clinic | Nomogram Vs Intra_Rad | Nomogram Vs Peri_Rad | Nomogram Vs Intra_Peri_Rad | Intra_Peri_Rad Vs Intra_Rad | Intra_Peri_Rad Vs Peri_Rad | Intra_Peri_Rad Vs Clinic | cohort |
| --- | --- | --- | --- | --- | --- | --- | --- |
| 0.0690425 | 0.14228 | 0.331297 |  | 0.14228 | 0.331297 | 0.0690425 | Train |
| 0.0947469 | 0.00600303 | 0.0582429 | 0.0940875105612652 | 0.0649028 | 0.696238 | 0.778922 | Test |

| 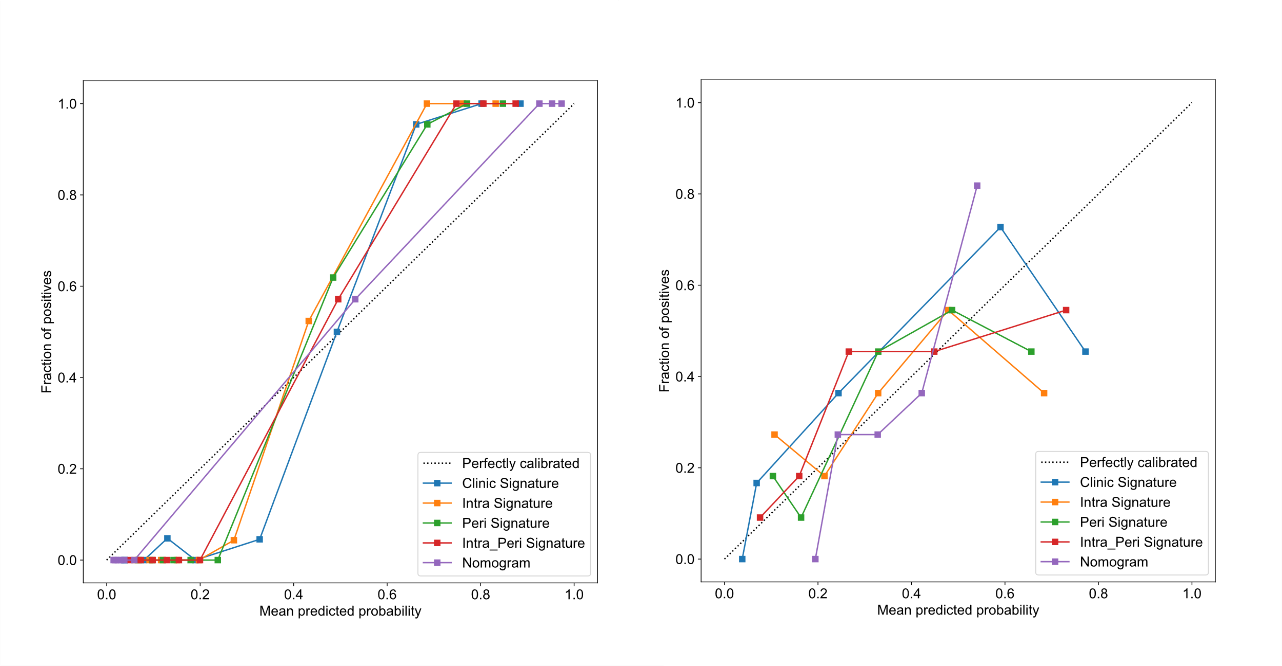  Figure S7: **The calibration curves in train and test cohort of different models** (Left: Training cohort, Right: Testing cohort) |
| --- |

Table S5: **P values of Hosmer-Lemeshow test in different signatures**

| Clinic Signature | Intra Signature | Peri Signature | Intra_Peri Signature | Nomogram | Cohort |
| --- | --- | --- | --- | --- | --- |
| 3.52702e-05 | 3.32309e-06 | 1.88341e-05 | 0.000326901 | 0.795245 | Train |
| 0.0342789 | 0.0336949 | 0.253092 | 0.261303 | 0.0925699 | Test |

| 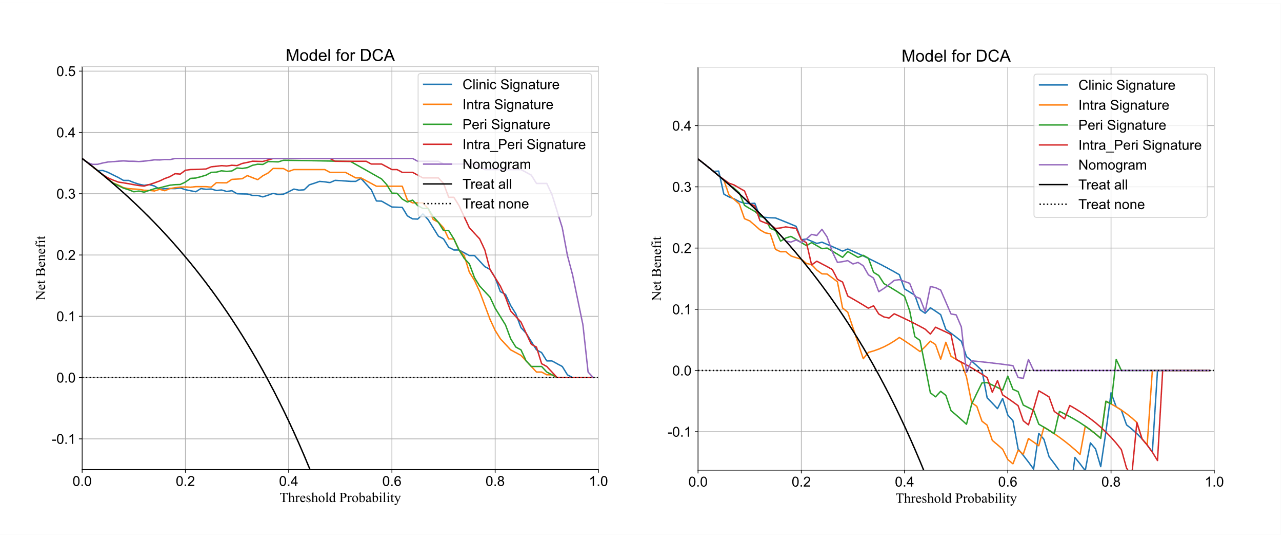  Figure S8: **Decision curve analyses on training(left) and testing cohort(right) of different models** |
| --- |

Table S6: **C-indexes of overall samples for survival function**

| Nomogram-Cox | Rad_Sig | Clinic_Sig | Cohort |
| --- | --- | --- | --- |
| 0.888 | 0.681 | 0.742 | Train |
| 0.899 | 0.648 | 0.717 | Test |
